# Dengue Virus Non-structural Protein 1 Modulates Infectious Particle Production via Interaction with the Structural Proteins

**DOI:** 10.1371/journal.ppat.1005277

**Published:** 2015-11-12

**Authors:** Pietro Scaturro, Mirko Cortese, Laurent Chatel-Chaix, Wolfgang Fischl, Ralf Bartenschlager

**Affiliations:** Department of Infectious Diseases, Molecular Virology, Heidelberg University, Heidelberg, Germany; National Institutes of Health, UNITED STATES

## Abstract

Non-structural protein 1 (NS1) is one of the most enigmatic proteins of the Dengue virus (DENV), playing distinct functions in immune evasion, pathogenesis and viral replication. The recently reported crystal structure of DENV NS1 revealed its peculiar three-dimensional fold; however, detailed information on NS1 function at different steps of the viral replication cycle is still missing. By using the recently reported crystal structure, as well as amino acid sequence conservation, as a guide for a comprehensive site-directed mutagenesis study, we discovered that in addition to being essential for RNA replication, DENV NS1 is also critically required for the production of infectious virus particles. Taking advantage of a *trans*-complementation approach based on fully functional epitope-tagged NS1 variants, we identified previously unreported interactions between NS1 and the structural proteins Envelope (E) and precursor Membrane (prM). Interestingly, coimmunoprecipitation revealed an additional association with capsid, arguing that NS1 interacts via the structural glycoproteins with DENV particles. Results obtained with mutations residing either in the NS1 *Wing* domain or in the *β-ladder* domain suggest that NS1 might have two distinct functions in the assembly of DENV particles. By using a trans-complementation approach with a C-terminally KDEL-tagged ER-resident NS1, we demonstrate that the secretion of NS1 is dispensable for both RNA replication and infectious particle production. In conclusion, our results provide an extensive genetic map of NS1 determinants essential for viral RNA replication and identify a novel role of NS1 in virion production that is mediated via interaction with the structural proteins. These studies extend the list of NS1 functions and argue for a central role in coordinating replication and assembly/release of infectious DENV particles.

## Author Summary

Dengue virus (DENV) is a major arthropod-borne human pathogen, infecting more than 400 million individuals annually worldwide; however, neither a therapeutic drug nor a prophylactic vaccine is currently available. Amongst the DENV proteins, non-structural protein 1 (NS1) is one of the most enigmatic, being required for RNA replication, but also secreted from infected cells to counteract antiviral immune response, thus contributing to pathogenesis. Despite its essential role at early stages of the viral replication cycle, the molecular determinants governing NS1 functions are unknown. Here, we used a combination of genetic, high-resolution imaging and biochemical approaches and found that NS1 additionally plays an important role for the production of infectious virus particles. By using a novel *trans*-complementation system with fully functional epitope-tagged NS1, we show that NS1 interacts with the structural proteins residing in the envelope of the virus particle. An NS1 variant retained in the endoplasmic reticulum still supported efficient DENV particle production, demonstrating that secretion of NS1 is dispensable for virion production. This study expands the list of functions exerted by NS1 for the DENV replication cycle. Given this multi-functional nature, NS1 appears to be an attractive target for antiviral therapy.

## Introduction

Dengue is the most prevalent arthropod-borne viral disease affecting around 400 million people worldwide and causing around 25,000 deaths per year [1]. Dengue virus (DENV) infections can lead to a wide range of clinical manifestations, ranging from asymptomatic to life-threatening dengue hemorrhagic fever and shock syndrome. However, in spite of its high medical relevance, no prophylactic vaccines or antiviral therapies are currently available and therefore a better understanding of the flavivirus life cycle is essential to promote the development of effective therapeutic regimens.

DENV has a single stranded RNA genome of positive polarity, encoding for a polyprotein that is co- and post-translationally processed into three structural proteins (capsid, prM, and envelope) and seven nonstructural proteins (NS1-NS2A-NS2B-NS3-NS4A-NS4B-NS5) [2]. After viral entry and release of the genomic RNA into the cytoplasm of infected cells, newly synthesized viral proteins induce massive remodeling of intracellular membranes, creating distinct intracellular structures where viral RNA replication and virion assembly take place [3,4]. Nucleocapsid formation, thought to occur in close proximity to replication sites, is likely accompanied by acquisition of a lipid envelope via budding into endoplasmic reticulum (ER) membranes enriched in the envelope protein E and prM [5,6], through as yet undefined mechanisms. Assembled virions, stored within ER stacks in highly ordered arrays, are then released from the cell via the conventional secretory pathway, where cleavage of the prM protein by furin, a protease residing in the *trans*-Golgi network (TGN), renders the viral particles infectious.

**Fig 1:**
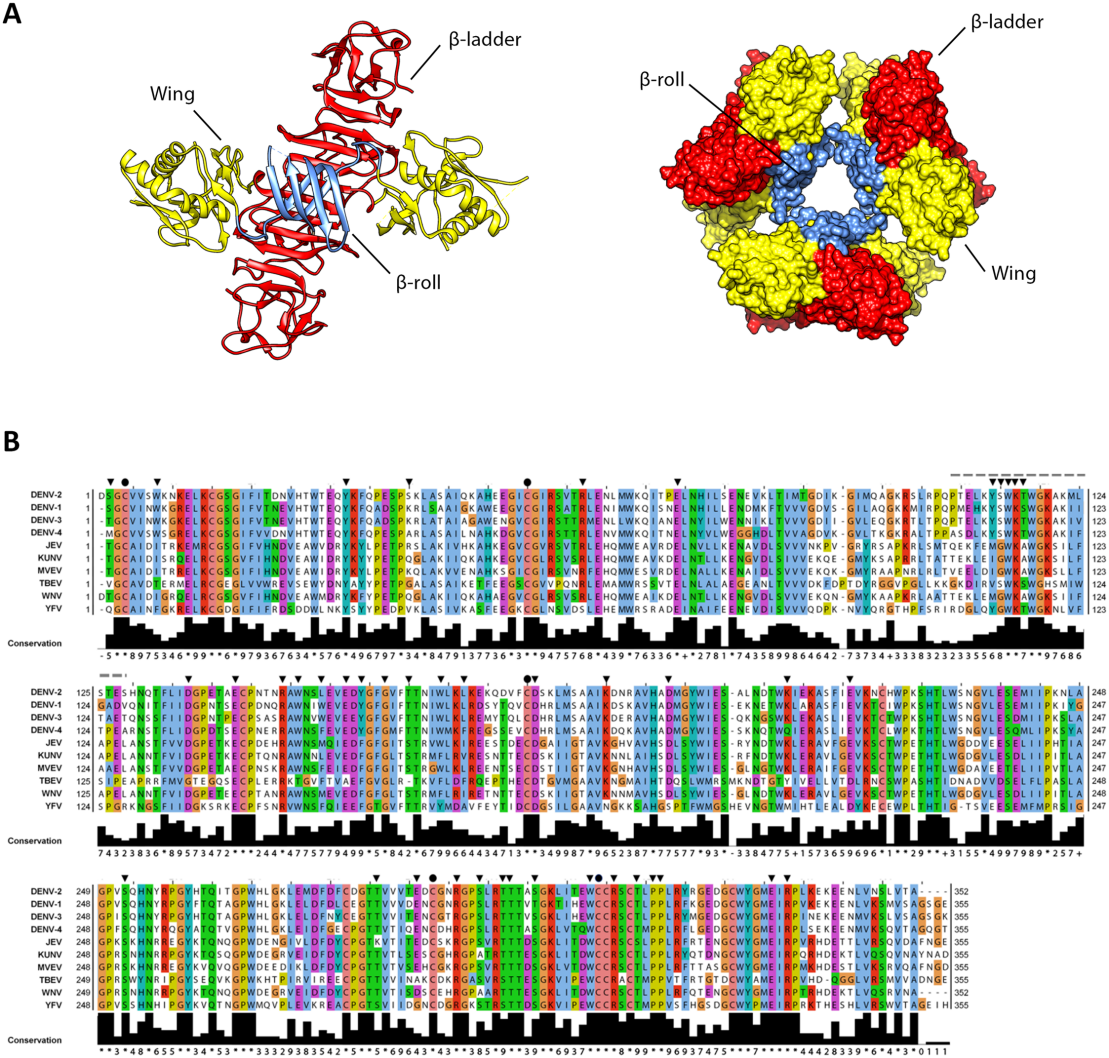
3D structure of NS1 and highly conserved residues targeted by site-directed mutagenesis. (**A**) *3D organization of the DENV NS1 dimer and hexamer*. The left panel shows a *ribbon* representation of the 3D crystal structure of the DENV NS1 dimer (Protein Data Bank [PDB] accession no. 4O6B) [9]. The *β-roll*, *Wing* and *β-ladder* domains are highlighted in blue, yellow and red, respectively. The right panel shows the 3D organization of the NS1 hexamer, with NS1 domains highlighted in the same color-code of the dimer. (**B**) Alignment of NS1 amino acid sequences of different DENV serotypes and related flaviviruses (JEV, Japanese encephalitis virus; KUNV, Kunjin virus; MVEV, Murray Valley Encephalitis virus; TBEV, Tick-Borne Encephalitis virus; WNV, West Nile virus; YFV, Yellow Fever virus). Residues are shown in different colors according to the *ClustalW* algorithm. The bottom line shows the amino acid conservation across different NS1 sequences. Black arrowheads and circles indicate residues targeted by site-directed mutagenesis with black circles indicating known cysteine residues engaged in disulfide bonds. The dashed line indicates the region that was not resolved in the 3D X-ray crystal structure.

Flavivirus NS1 is a multifunctional 48-kDa glycoprotein that is translocated into the ER lumen co-translationally. Within the ER, NS1 promptly dimerizes upon addition of high-mannose carbohydrates [7], and is targeted to three destinations: the viral replication sites, the plasma membrane and the extracellular compartment. The majority of secreted NS1 is a soluble, proteolipid particle forming an open-barrel hexameric shell with a central channel occupied by lipids [8]. The three-dimensional high-resolution structure of the DENV NS1 dimer was recently solved by X-ray crystallography [9], providing valuable insights into the complex NS1 fold (Fig 1). The dimer contains three domains: first, a small *β-roll* domain formed by two intertwined β-hairpins; second, a *Wing* domain, composed of an α/β subdomain and a discontinuous connector that sits against the *β-roll*; third, a *β-ladder* domain, formed by 18 antiparallel β-strands (9 contributed by each monomer) assembled in a continuous β-sheet that runs along the whole length of the dimer (Fig 1A, left panel). The protrusion created by the *β-roll* and the connector subdomain renders one side of the dimer hydrophobic, and has been proposed to face the ER membrane and to interact with other transmembrane viral proteins [9,10]. Conversely, within the NS1 hexamer, the *β-roll* faces the interior of the lipoparticle, where it associates with the central lipid core (Fig 1A, right panel). On the opposite side of the *β-roll*, both in the dimeric and the hexameric form, the distal tips of the *β-ladder* and the *Wing* domain loops point outward, and are therefore exposed to the solvent.

Secreted NS1 as well as NS1 residing on the plasma membrane and within cells, plays important roles in immune evasion via binding to complement proteins and modifying or antagonizing their functions [11–14]. Besides its immune evasive functions, NS1 modulates early events in viral RNA replication, was shown to co-localize with double strand RNA (dsRNA) and to interact with NS4B [10,15–17]. Indeed, deletion of NS1 from the viral genome completely abrogates replication, but ectopic expression of NS1 *in trans* can efficiently rescue NS1-deleted (ΔNS1) viruses [18–21].

Because of its essential role early in RNA replication, genetic studies have thus far provided limited information on the molecular determinants of NS1 responsible for the viral replication cycle and did not investigate possible functions of the protein for assembly and release of infectious virus particles. By using a combination of genetic, high-resolution imaging and biochemical approaches we discovered a novel role of NS1 for the production of infectious DENV particles that is linked to NS1 interaction with the structural proteins, but independent from NS1 secretion.

## Results

### Identification of critical NS1 determinants required for DENV replication

**Fig 2:**
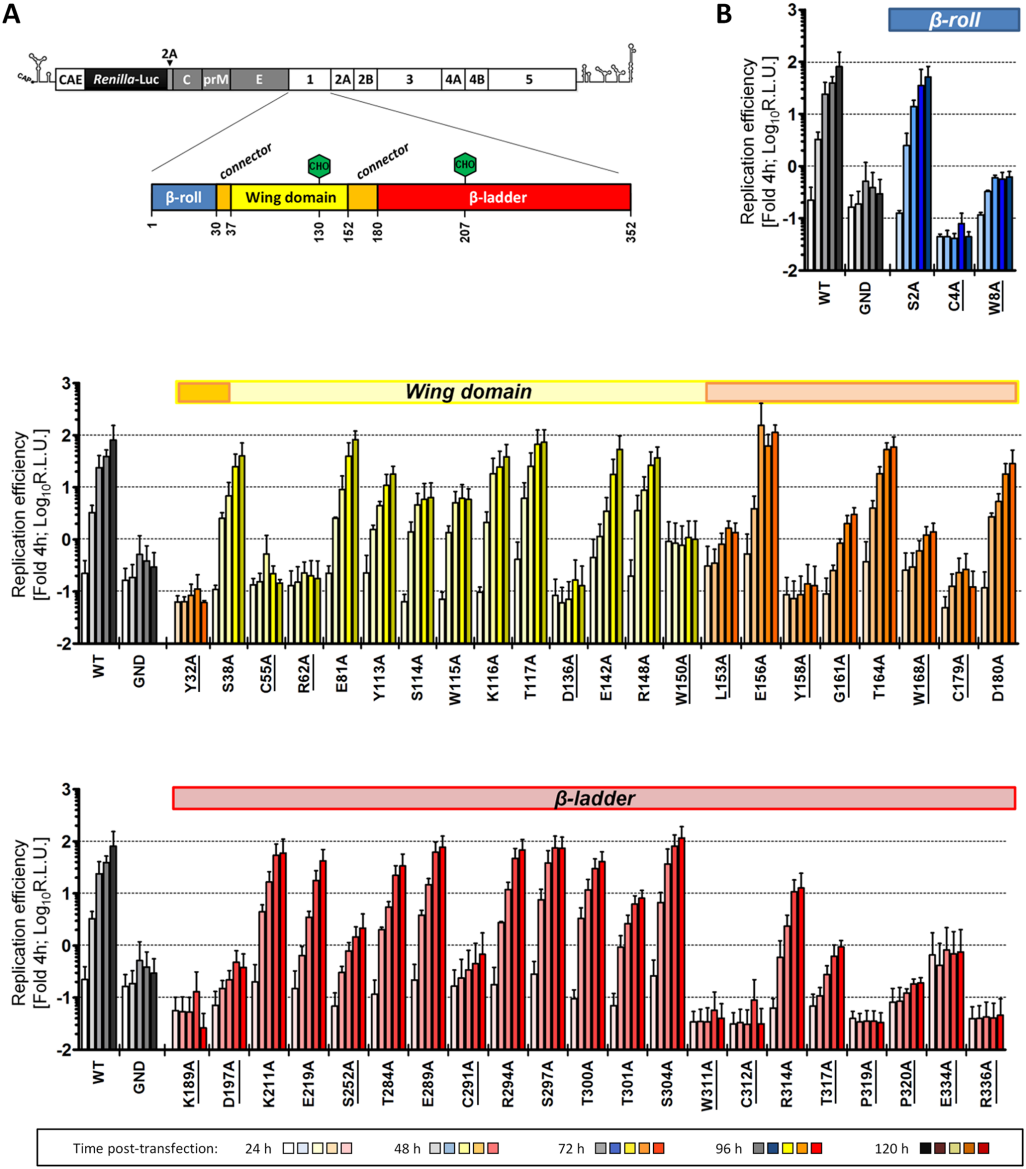
Effect of NS1 Alanine substitutions on DENV replication. (**A**) *Schematic representation of the DENV reporter virus genome and NS1 protein domains*. The full-length luciferase reporter DENV genome (DVR2A) is shown at the top, with the 5’ and 3’ NTRs depicted with their putative secondary structures. Polyprotein cleavage products are separated by vertical lines and labeled as specified in the introduction. A *Renilla luciferase* coding sequence was inserted in-between the capsid cyclization sequence (CAE) and the *Tosea asigna* virus 2A cleavage site that ensures proper processing after polyprotein synthesis. NS1 protein domains shown below are indicated as described in Fig 1. Glycosylation sites are shown with green hexagons, while the *β-roll*, *Wing* and *β-ladder* domains are shown in blue, yellow and red, respectively. Two *connector* sub-domains within the *Wing* domain are shown in orange. (**B**) *Replication kinetics of DENV NS1 mutants*. VeroE6 cells were electroporated with *in-vitro* transcribed luciferase virus RNAs containing NS1 mutations specified at the bottom. Cells were lysed 4, 24, 48, 72, 96 and 120 h after transfection and luciferase activity in cell lysates was determined. Data were normalized to the 4h-value that reflects transfection efficiency. The background of the assay was determined with the active site NS5 polymerase mutant (GND). Colored lines on the top of each panel refer to the color-coded representation of NS1 protein domains as shown in (A). NS1 mutants severely impaired in viral RNA replication (normalized luciferase activity ≤0) are underlined. For ease of comparison, data obtained with WT and GND are repeated in the left of each panel. Mean values and standard deviations of three independent experiments are shown.

Sequence analysis and visual inspection of the recently solved three-dimensional crystal structure [9] of NS1 were performed to assess the degree of conservation of amino acid residues and to identify the most relevant positions to be targeted by site-directed mutagenesis (Fig 1B). Based on their distribution within the NS1 dimer and their relative conservation across the *Flavivirus* genus, we selected 46 residues for alanine scanning mutagenesis, including five invariant cysteine residues (C4, C55, C179, C291, C312), recently shown to be engaged in disulfide bonds and playing an essential role in stabilizing the protein fold [9,22]. To dissect the impact of each individual mutation on the different steps of the viral replication cycle, we assessed viral RNA replication and virus spread by taking advantage of a DVR2A luciferase reporter virus genome (Fig 2A). VeroE6 cells were electroporated with *in vitro* transcripts of *wild-type* (WT) or a given NS1 mutant and viral replication was assessed by luciferase activity 24, 48, 72, 96 and 120 h later (Fig 2B). Additionally, a replication-deficient NS5 mutant (GND) with a lethal mutation affecting the RNA-dependent RNA polymerase activity was included as negative control. Based on the replication phenotypes, half of the NS1 mutants displayed only minor defects or replicated comparably to WT (Table 1). Conversely, 23 mutations, including those affecting cysteine residues engaged in disulfide bonds, severely or completely blocked viral RNA replication (Fig 2B and Table 1, underlined mutants). Most of these mutations clustered on the core of the *Wing* and *β-ladder* domain, affecting residues that point towards the *β-roll* (S1 Fig), which has been proposed to face the ER membrane [9]. Interestingly, in close proximity to the previously reported di-amino acid motif (N10-K11) suggested to mediate interaction with NS4B and association with ER membranes [10], a mutation targeting W8 within the NS1 *β-roll* domain completely abrogated viral RNA replication. Similarly, mutations within the *greasy finger* loop of the *β-ladder*, namely Y158A and G161A, resulted in a lethal phenotype as already shown for alanine substitutions at residues G159 and F160 [9]. Altogether, these results provide a comprehensive map of molecular determinants within NS1 essential for viral RNA replication, highlighting an important role for selected residues of the *β-roll* and *β-ladder* domains, clustering on hydrophobic protrusions within the NS1 dimer structure (S1 Fig).

**Table 1:** Effect of NS1 alanine substitutions on DENV reporter virus replication.

Mutation	Replication Efficiency 48h (fold-of-4h; normalized to WT)	Mutation	Replication Efficiency 48h (fold-of-4h; normalized to WT)
WT	1.00 ± 0.00	R168A	0.08 ± 0.08
GND	0.03 ± 0.01	C179A	0.10 ± 0.12
S2A	1.02 ± 0.52	D180A*	1.69 ± 1.59
C4A	0.02 ± 0.02	K189A	0.04 ± 0.07
W8A	0.17 ± 0.08	D197A	0.10 ± 0.11
Y32A	0.03 ± 0.02	K211A	3.07 ± 3.37
S38A	1.37 ± 0.94	E219A	0.29 ± 0.08
C55A	0.09 ± 0.07	S252A	0.18 ± 0.16
R62A	0.06 ± 0.04	T284A	1.14 ± 0.86
E81A	1.28 ± 0.49	E289A	2.07 ± 1.43
Y113A	0.80 ± 0.34	C291A	0.23 ± 0.37
S114A*	0.63 ± 0.20	R294A	1.59 ± 1.20
W115A*	0.63 ± 0.10	S297A	2.12 ± 0.50
K116A	0.93 ± 0.18	T300A	0.94 ± 0.14
T117A	1.12 ± 0.55	T301A*	0.26 ± 0.06
D136A	0.02 ± 0.02	S304A	1.94 ± 0.59
E142A	0.22 ± 0.08	W311A	0.01 ± 0.01
R148A	0.64 ± 0.20	C312A	0.01 ± 0.01
W150A	0.11 ± 0.13	R314A	0.17 ± 0.12
L153A	0.09 ± 0.09	T317A	0.03 ± 0.01
E156A	0.78 ± 0.20	P319A	0.01 ± 0.01
Y158A	0.02 ± 0.03	P320A	0.04 ± 0.06
G161A	0.06 ± 0.02	E334A	0.12 ± 0.18
T164A	0.90 ± 0.29	R336A	0.01 ± 0.01

The replication level of a given mutant relative to the wild-type (WT) is displayed. Replication was determined by measuring luciferase activity 48 h post-transfection and normalizing to the 4 h value that reflects transfection efficiency. Values represent mean ± standard deviation of three independent experiments. NS1 mutants with severe defects in viral RNA replication are underlined, while asterisks (*) indicate mutants with selective defects in virus assembly or release.

### Identification of NS1 as a critical factor for infectious particle production

**Fig 3:**
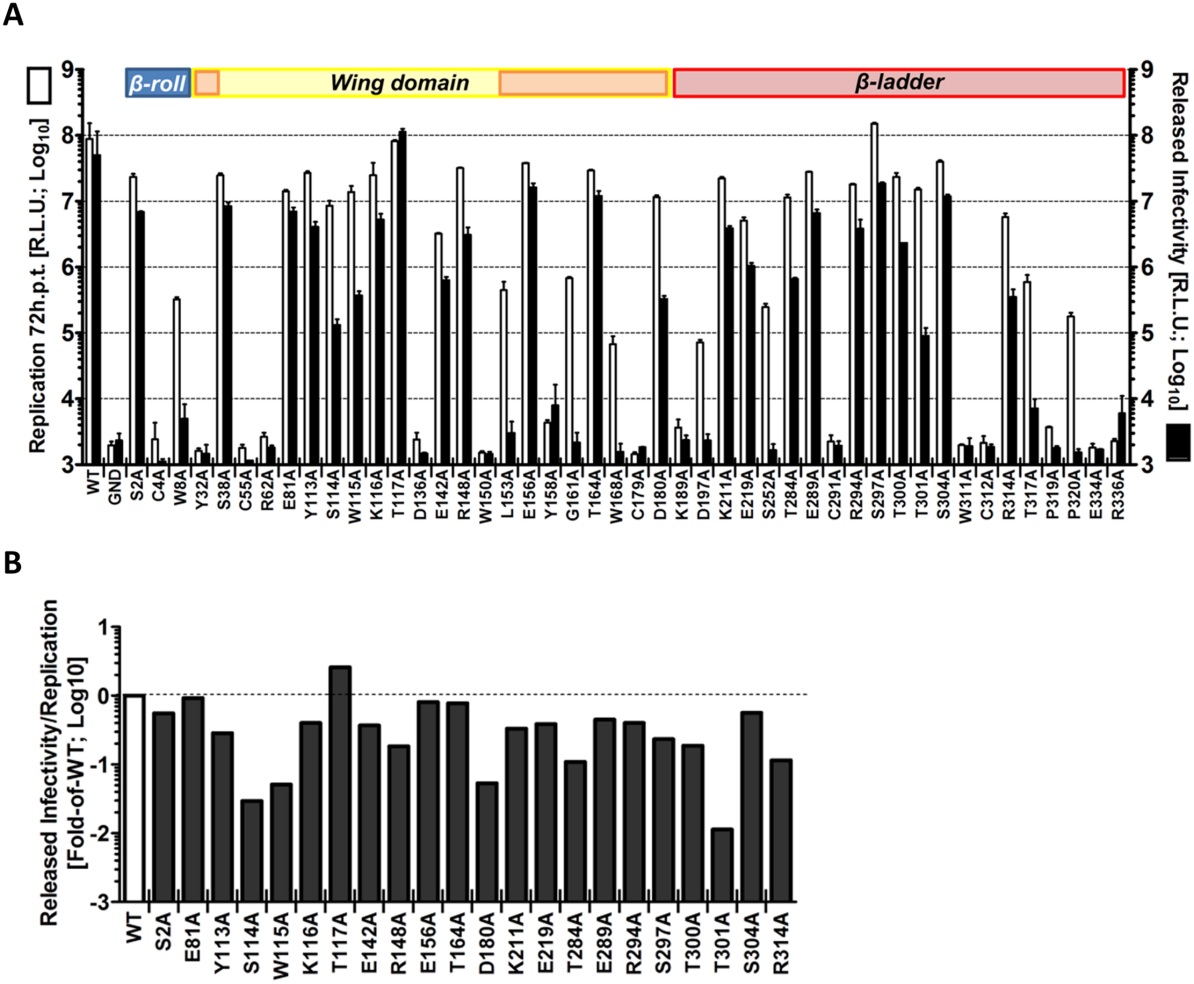
Effect of mutations in NS1 on the production of infectious DENV particles. (**A**) *Released infectivity from DVR2A NS1 mutant-transfected cells*. VeroE6 cells were electroporated with DENV genomes containing NS1 mutations specified at the bottom. Seventy-two hours post-electroporation, supernatants containing infectious virus were used to infect naïve VeroE6 cells and luciferase activity measured 48 h later (black columns). Luciferase activity in the lysates 72 h post-electroporation reflecting replication is also shown (white columns). The background of the assay is determined with the active site NS5 polymerase mutant (GND). Mean values and standard deviations of three independent experiments are shown. Colours at the top of the panels refer to NS1 protein domains as described in Fig 2A. (**B**) *Efficiency of virus production relative to replication fitness*. To calculate specific defects in infectious particle production, data presented in (A) are shown as ratio of released infectivity/viral replication, and expressed as fold of wild-type (WT). Note that only NS1 mutants with viral RNA replication above input values were considered (cf. Fig 2B; replication > Log_10_0).

To determine the impact of each mutation on the production of infectious virus particles, culture supernatants of transfected cells (Fig 2B) were harvested 72 h after transfection and used to infect naïve VeroE6 cells. Virus production was determined by luciferase assay 48 h later (Fig 3A). This experiment revealed a group of mutations (S114A, W115A, D180A, T301A) with minor effects on RNA replication, but massive impairment of virus production (up to ~2.5 Log_10_ reduction compared to WT) (Fig 3B). Noteworthy, a mutation targeting T117 within the unresolved stretch of the *Wing* domain and in close proximity to S114 and W115, slightly enhanced particle production. Altogether, these results suggest a previously undiscovered role of NS1 for the production of infectious DENV particles.

**Fig 4:**
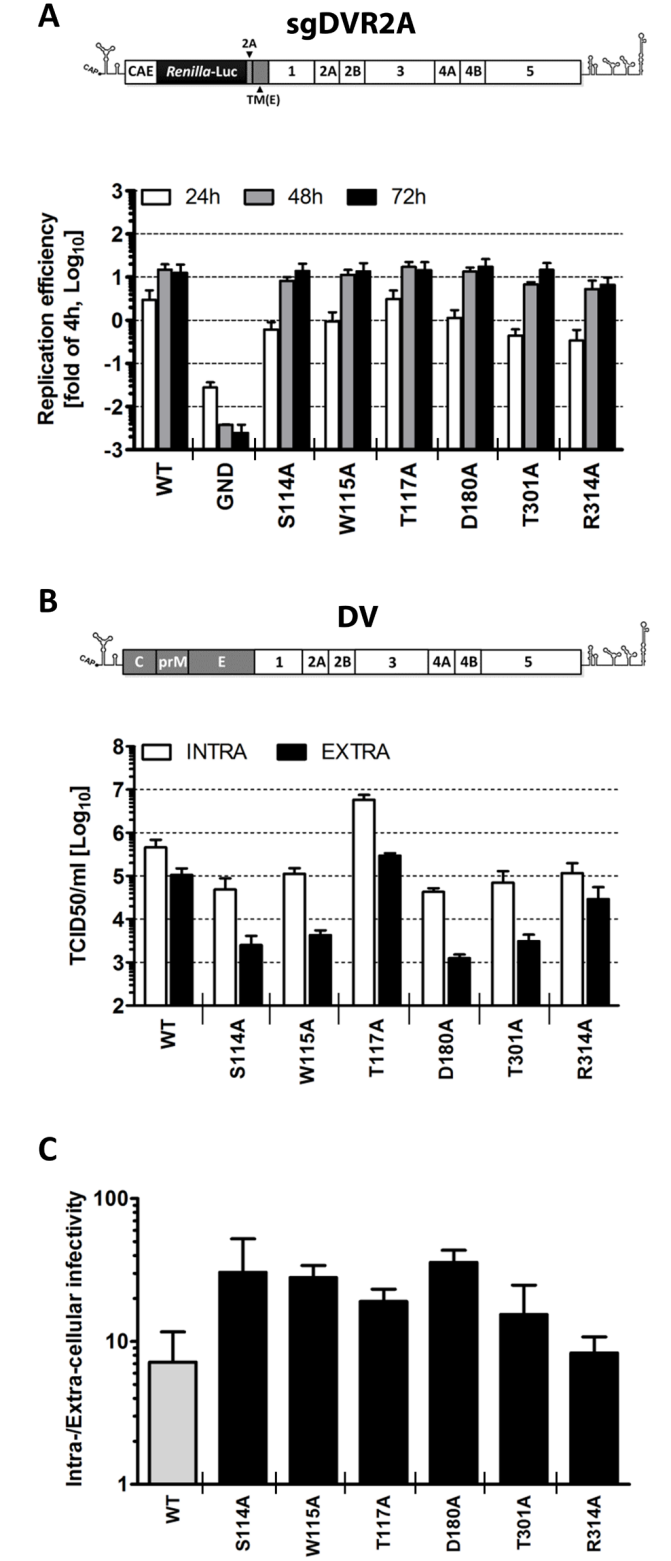
Characterization of selected NS1 mutants reveals specific defects in infectious particle production. **(A)** *Replication kinetics of NS1 mutants in the context of a sub-genomic replicon*. Schematic representation of the sub-genomic reporter replicon (sgDVR2A) is shown at the top. It is derived from the DV2 full-length genome by insertion of a *Renilla luciferase* coding sequence in-between the capsid cyclization sequence (CAE) and the *Tosea asigna* virus 2A cleavage site. The last 24 amino acid residues of the envelope coding region (TM) at the N-terminus of NS1 ensure proper membrane topology of the polyprotein after synthesis. Selected NS1 mutations affecting virus production were inserted into sgDVR2A, and *in vitro* transcribed RNAs were electroporated into VeroE6 cells. Luciferase activity was measured in the lysates 4, 24, 48 and 72 h later. Values are expressed as fold of the 4h-value which reflects transfection efficiency. The background of the assay is determined with the active site NS5 polymerase mutant (GND). Columns represent mean and standard deviations of three independent experiments. (**B**) *Intra- and extra-cellular infectivity titers of NS1 mutants*. The structure of the full-length DENV genome used for this analysis is shown at the top (DV). NS1 mutations specified at the bottom were inserted into DV and in vitro transcripts derived therefrom were transfected into Huh7 cells and used for determination of intra- and extracellular infectivity. Seventy-two hours post electroporation, supernatants and cell lysates were subjected to 3 freeze and thaw cycles and titers were determined by TCID_50_ assay using Huh7 cells and an envelope protein-specific MAb. Mean values and standard deviations of three independent experiments are shown. (**C**) Evidence that NS1 mutations affect, in part, release of infectious DENV particles. To determine the relative contribution of defects in virus assembly and virus release, data presented in (B) were used to calculate the ratio of intra- vs. extra-cellular infectivity.

Next we wanted to corroborate this observation and rule out that impaired virus production was an indirect consequence of diminished replication fitness rather than a specific defect in assembly or release of viral progeny. To this end, we assessed the impact of these mutations on RNA replication in the context of a sub-genomic reporter replicon (sgDVR2A) that does not support virus production, thus measuring replication independent from a possible contribution of virus spread (Fig 4A). In this and all subsequent analyses we focused on mutants with selective alterations of virus production (S114A, W115A, D180A, T301A; Fig 3B) in order to avoid possible indirect effects resulting from impaired replication fitness (Fig 3B). Therefore, NS1 mutants with strong replication defects were excluded (Table 1). Moreover, since several NS1 mutants with a defect in virus production were slightly impaired in RNA replication we included as control the NS1 mutation R314A that caused minimal defect in replication, but did not affect virus production (Table 1). Furthermore, mutant T117A was included in the analysis because of its increased capacity to produce infectious DENV particles and its close proximity to some of the sites where mutations caused a selective reduction of virus production. VeroE6 cells were transfected with *in vitro* transcripts of the replicon constructs and replication was measured 24, 48 and 72 h later (Fig 4A). While at early times post transfection we observed a moderate reduction in luciferase activity for S114A, W115A, D180A and T301A compared to WT (2 to 5-fold; 24 h.p.t.), all mutants replicated comparably at 48 and 72 h.p.t., arguing for a minor contribution of RNA replication to the observed reduction in infectious particle production. As already observed in the context of the full-length reporter virus, the T117A mutant exhibited a replication profile comparable to WT also within the subgenomic sgDVR2A replicon whereas R314A exhibited an overall decrease in replication fitness, with a 50% to 75% reduction at any time point. Collectively, these results demonstrate a selective defect for a sub-group of NS1 mutants in assembly and/or release of virus particles.

To investigate further the phenotype of the NS1 mutants with respect to the production of infectious intra- and extracellular virus particles, and to corroborate these observations in human cells, we determined the infectivity profiles of each mutant in the context of a full-length DENV genome transfected into human hepatoma Huh7 cells. Three days post-transfection, titers of infectious virus released into the culture media or contained in cells were determined by limiting dilution assay with naïve cells. The results shown in Fig 4B demonstrate that the amounts of intra- and extracellular infectivity were altered in all mutants, albeit to very different degrees. In agreement with the results obtained with the reporter virus genome, we found that alanine substitutions at residues S114, W115, D180 and T301 reduced extracellular infectivity titers up to 100-fold, confirming an essential role of these amino acid residues in NS1 for particle production. Interestingly, the amounts of intracellular virus particles were also reduced 5- to 10-fold. While this impairment argued for a defect of the NS1 mutants in virus assembly or maturation, the higher reduction of extracellular virus titers suggested an additional effect on particle release as inferred from the ratio of intra- to extracellular virus titers and comparison with the WT (Fig 4C). The R314A mutation reduced the virus titer only ~3.5-fold and did not affect the ratio of intra- to extracellular infectivity, consistent with a subtle effect on assembly or virus maturation (Fig 4C). Interestingly, the T117A mutant produced 12-fold more intracellular virus than WT, concomitant with a ~5-fold higher titer of extracellular virus particles, indicating accelerated assembly or infectivity maturation and reduced virus particle release. In conclusion, these results suggest that alanine substitutions at position S114, W115, T117, D180 or T301 of NS1 alter the production of infectious virus, supporting the notion that NS1 is a critical determinant for assembly or release of infectious virus particles.

### NS1 secretion is dispensable for DENV virus entry, RNA replication and particle release

NS1 accumulates in extracellular fluids as a homo-hexamer with a lipidic core [8,23,24] and besides its immune evasive functions [11–13] was shown to enhance virus attachment upon entry [25]. In addition, some studies hypothesized a link between NS1 secretion and virus assembly or release [26]; however the lack of genetic tools in those days allowing the selective block of NS1 secretion in the context of a complete replication cycle precluded any functional investigation.

**Fig 5:**
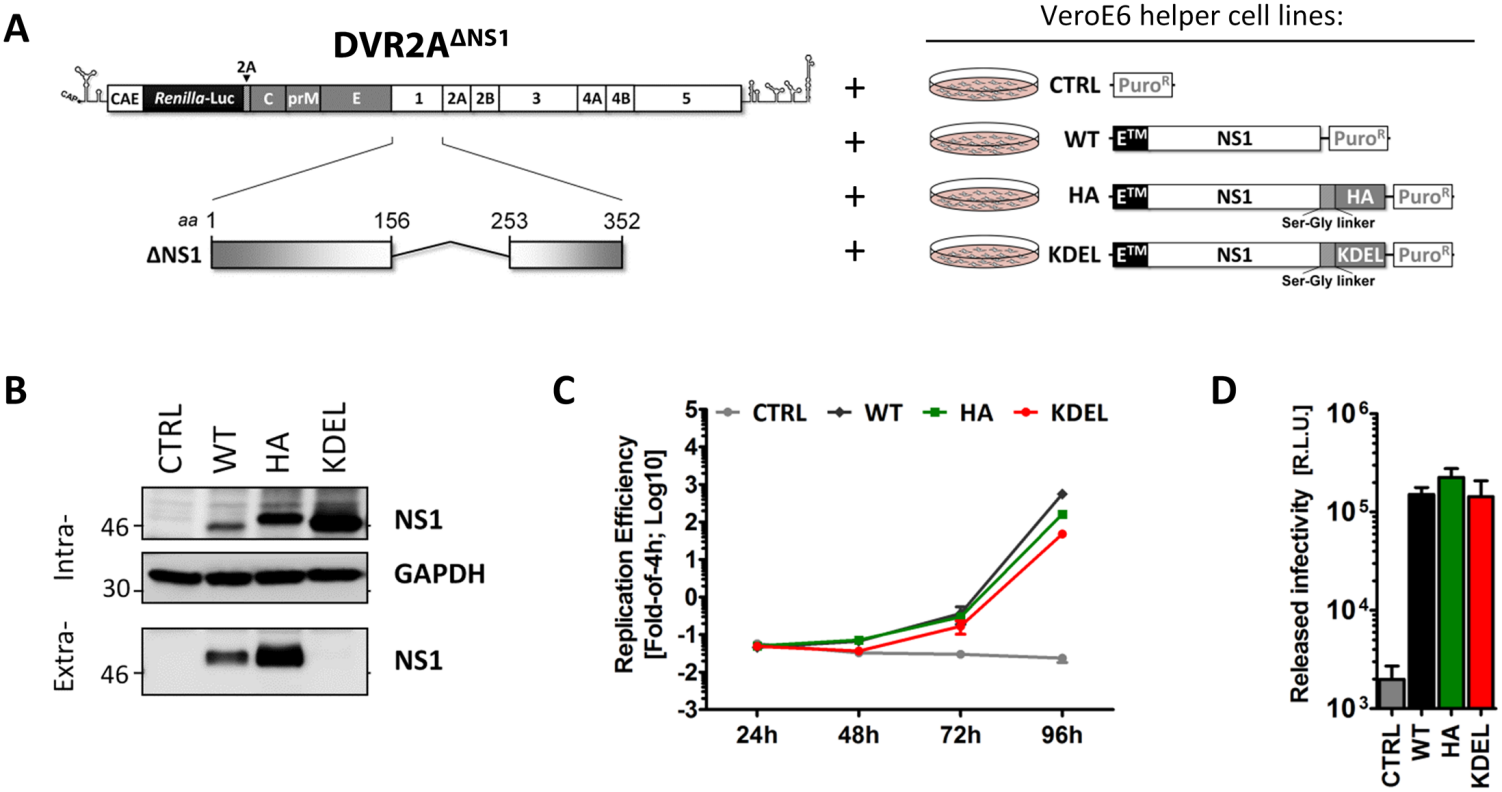
NS1 secretion is dispensable for the production of infectious DENV particles. (**A**) *Schematic representation of the delta-NS1 DENV genome and helper cell lines employed*. A deletion comprising 97 codons in NS1 (Δ156–253) was introduced into the full-length DVR2A genome (DVR2A^ΔNS1^). VeroE6 helper cells (right panel) containing the stably integrated pWPI expression vector without insert (CTRL), or encoding full-length *wild-type* NS1 (WT), or NS1 C-terminally fused with an HA epitope (HA) or NS1 C-terminally fused with a KDEL ER retention motif (KDEL), were generated by transduction with lentiviral vectors as described in materials and methods. (**B**) *Intra- and extra-cellular NS1 protein levels of VeroE6 helper cell lines*. Cell lysates or clarified supernatants were analyzed by immunoblotting, using the antibodies indicated on the right. The positions of molecular weight markers (kDa) are shown on the left. (**C-D**) *Replication efficiency and released infectivity of the ΔNS1 genome trans-complemented with NS1 variants*. (C) *In-vitro* RNA transcripts of DVR2A^ΔNS1^ were electroporated into VeroE6 helper cell lines expressing NS1 variants specified in the top and replication efficiency was determined in cell lysates at 4, 24, 48 and 72 h after transfection. (**D**) Clarified supernatants containing infectious particles were harvested 72h.p.t. and used to infect each respective naïve VeroE6_helper cells. Luciferase activity in the lysates was measured 48h later (right panel). The background of the assay was determined by infecting näive VeroE6 cells lacking exogenous NS1 (CTRL) and therefore not supporting DVR2A^ΔNS1^ replication. The dashed line indicates the limit of detection of the assay.

Prior studies have shown that YFV, KUNV and WNV mutants lacking NS1 do not replicate, but can be rescued when NS1 is complemented *in trans* by ectopic expression of the full-length protein [17,18,21,27]. To elucidate the possible role(s) of NS1 secretion in the DENV replication cycle, we utilized a similar approach and generated a DENV genome containing a 97 amino acids in-frame deletion within the NS1 gene (DVR2A^ΔNS1^). This mutant retained the N-terminal 156 and the C-terminal 99 residues, respectively (Fig 5A, left panel). In parallel, we engineered a set of helper VeroE6 cell lines, constitutively expressing different NS1 variants after lentiviral transduction of expression vectors containing the complete NS1 coding region (NS1^WT^) or the empty pWPI vector (CTRL) that served as positive and negative controls, respectively. Furthermore, we engineered C-terminally tagged variants carrying a HA-affinity epitope (NS1^HA^) or the well-described KDEL motif (NS1^KDEL^), responsible for retrieval of ER luminal proteins from the Golgi apparatus by retrograde transport (Fig 5A, right panel). Correct protein expression and secretion of each NS1 variant was confirmed by western-blotting (Fig 5B). As expected, NS1^HA^ had a slower electrophoretic mobility than the WT. Furthermore, both NS1^WT^ and NS1^HA^ were readily detected in the culture media, while NS1^KDEL^ was effectively retained in the ER.

Next, we assessed rescue of viral RNA replication and particle production by the NS1 variants by using transfection of the DVR2A^ΔNS1^ genome into each helper cell line. As shown in Fig 5C, the ΔNS1 genome was able to replicate in NS1^WT^, NS1^HA^ and NS1^KDEL^ helper cells, while no luciferase activity could be detected in CTRL cells lacking NS1. Of note, when each naïve helper cell line was infected with virus-containing culture fluids harvested 72 h.p.t, comparable levels of luciferase activity were detected in all conditions, indicating that C-terminally HA-tagged NS1 is fully functional (Fig 5D). Importantly, the rescue of particle production by NS1^KDEL^ shows that secretion of NS1 is dispensable for infectious DENV particle production.

**Fig 6:**
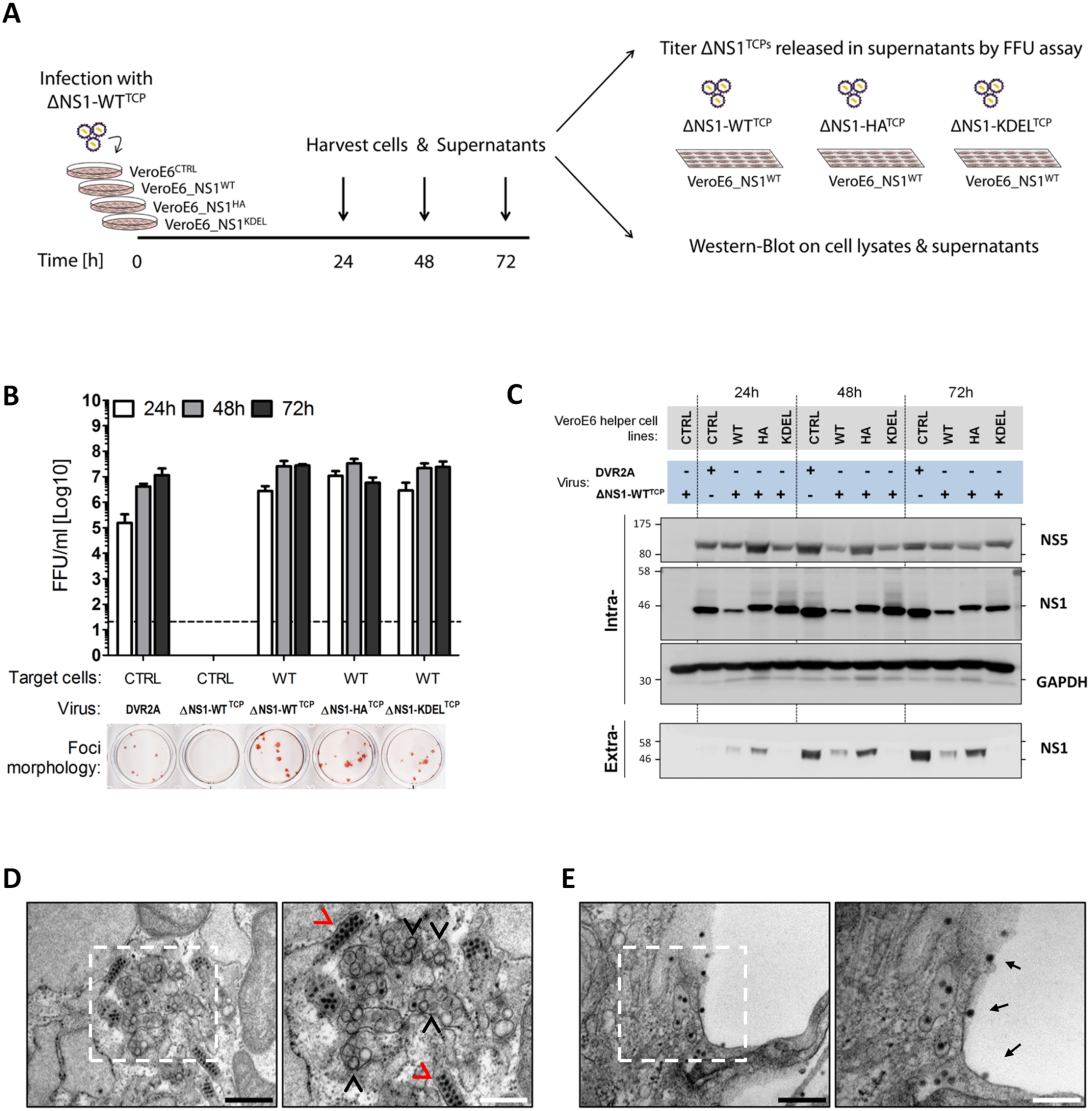
Secretion kinetics and ultrastructural characterization of ΔNS1^TCP^-infected helper cell lines. (**A**–**B**) *Kinetics of DVR2A* ^*ΔNS1*^ *TCPs secretion from helper cell lines*. (A) Schematic representation of the experimental setting. DVR2A^ΔNS1^ TCPs produced in VeroE6_NS1^WT^ cells (ΔNS1-WT^TCP^) were used to infect (MOI = 1) control or helper cell lines expressing different forms of NS1. Culture supernatants were collected 24, 48 and 72 h later, and virus titers were determined by Focus forming unit (FFU) assay on VeroE6_NS1^WT^ cells, using an E-specific mouse monoclonal antibody. Cell lysates and culture supernatants were analyzed by western blot. (B) FFU titers were determined as specified in panel (A). As reference, naïve VeroE6 cells were infected with DVR2A. The dashed line indicates the limit of detection of the assay. The lower panel shows representative images of foci morphologies of each trans-complemented NS1 variant. (**C**) *Expression levels of intra- and extra-cellular NS1 in DVR2A* ^*ΔNS1*^ *TCP infected cells*. NS1 expression and secretion were evaluated by western-blotting using cell lysates (Intra-) or clarified supernatants (Extra-) from (B) and NS1-, NS5- or GAPDH specific antibodies. (**D**, **E**) *Ultrastructural characterization of cells infected with DVR2A* ^*ΔNS1*^ *TCPs*. VeroE6_NS1^HA^ cells were infected with 1 MOI of DVR2A^ΔNS1^ TCPs. Forty-eight hours later, cells were fixed, processed and analyzed by transmission electron microscopy as described in materials and methods. Representative images of the perinuclear area (D) or plasma membrane (E) of infected cells are shown. Black arrowheads indicate Vesicle packets (VPs), red arrowheads indicate virion bags. Electron-dense virus particles in proximity to or at the plasma membrane are indicated with black arrows. Boxed areas on the left panels are shown at higher magnification on the right. Black or white scale bars represent 500 or 200 μm, respectively.

To address the relative efficiency of trans-complementation upon virus infection, NS1^WT^-, NS1^HA^- and NS1^KDEL^-expressing VeroE6 cells were infected with trans-complemented DVR2A^ΔNS1^ particles (ΔNS1^TCP^), produced in VeroE6_NS1^WT^ cells. Culture fluids were harvested 24, 48 and 72 h later and the amounts of produced particles were determined by focus-forming unit (FFU) assay on NS1^WT^ cells (Fig 6A). Consistent with the luciferase assay data, ΔNS1^TCP^ did not produce infectious virus on CTRL cells that do not express NS1, confirming that this mutant fails to replicate in the absence of NS1. Titers of infectious ΔNS1^TCP^ particles released from VeroE6_NS1^WT^, NS1^HA^ or NS1^KDEL^ cells were higher than DVR2A *wild-type* infection on CTRL cells at early times post-infection (24 and 48 h p.i.), with no appreciable differences observed at later time points (72 h p.i.) (Fig 6B). Interestingly, while none of the trans-complemented TCPs gave rise to clearly visible plaques as previously reported [17], DENV-containing foci detected by immunostaining were larger than those produced by the full-length *wild-type* virus (Fig 6B, lower panel).

Additionally, lysates and culture supernatants of infected cells were harvested 24, 48 and 72 h p.i. to evaluate protein expression and secretion upon ΔNS1^TCP^ infection. Under these conditions, virus replication in the various helper cell lines was comparable as judged by the intracellular protein levels of NS5 (Fig 6C; “Intra“). Furthermore, secretion profiles of HA- WT- and KDEL-tagged NS1 resembled those of uninfected cells, with the latter being efficiently retained intracellularly also upon DENV infection (Fig 6C; “Extra“).

To unequivocally confirm that the ΔNS1^TCP^ system fully recapitulates wild-type DENV infections, we additionally investigated the ultrastructural morphology of NS1^HA^ helper cells upon infection with DVR2A^ΔNS1^ TCPs by using transmission electron microscopy. In agreement with the replication and infectious particle production data, NS1^HA^ cells infected with DVR2A^ΔNS1^ TCPs contained the characteristic membrane invaginations which have been proposed to represent viral replication factories (vRFs) [5,6] and electron-dense virus particles forming regular arrays within the ER (Fig 6D). Moreover, a large number of virus particles were observed on the plasma membrane or accumulating within the extracellular space between adjacent cells (Fig 6E). These structures were absent in both uninfected NS1^HA^ cells and ΔNS1^TCP^-infected CTRL cells (S2 Fig).

In conclusion, these results demonstrate full functionality of HA-tagged and ER-retained NS1 supporting both DENV RNA replication and production of infectious virus particles.

### NS1 associates with the viral envelope glycoproteins

**Fig 7:**
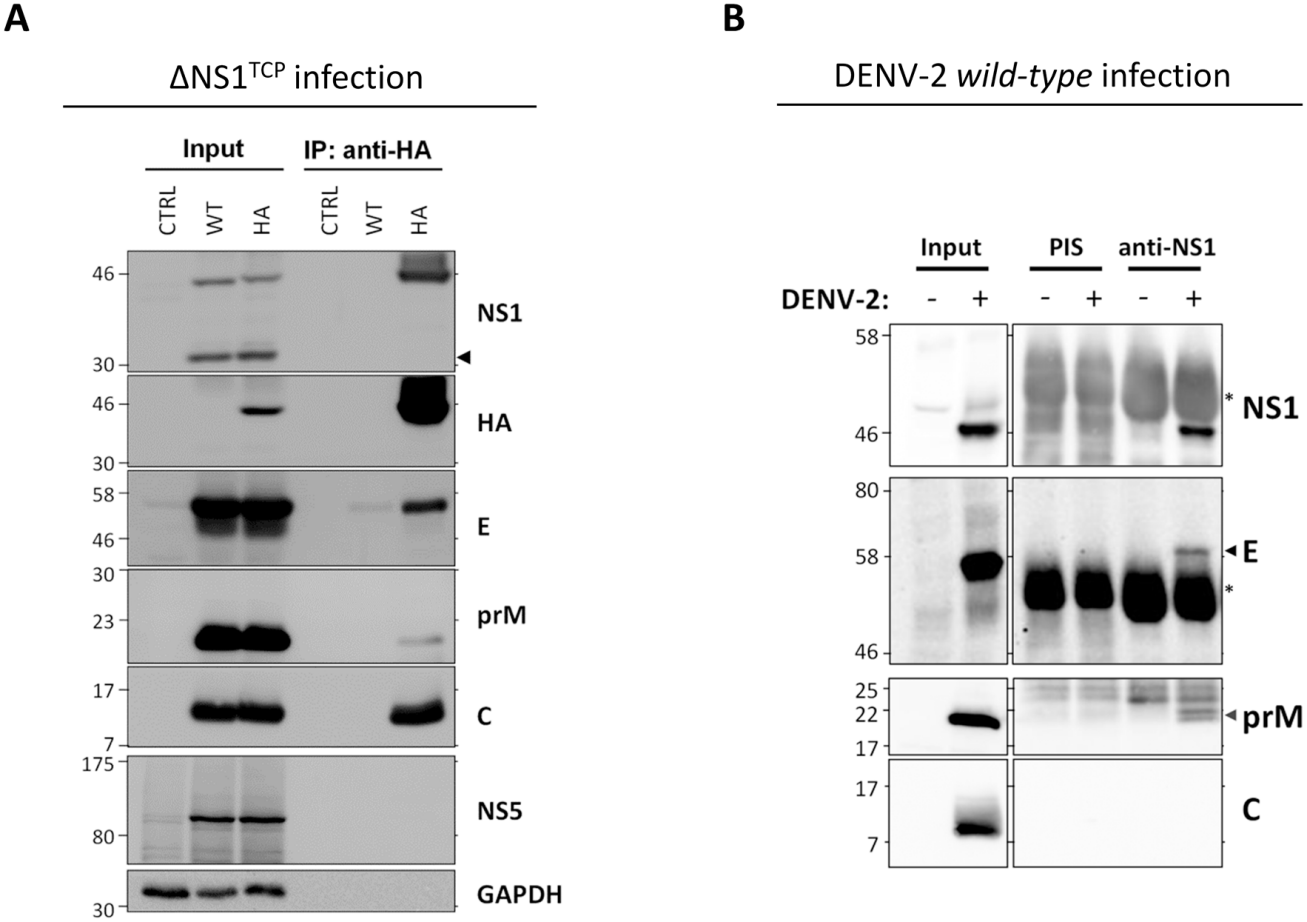
NS1 interacts with the structural proteins. (**A**) *Capsid*, *prM and Envelope proteins co-immunoprecipitate with NS1*. Naïve VeroE6 (CTRL), VeroE6_NS1^WT^ (WT) or VeroE6_NS1^HA^ (HA) cells were infected with 1 MOI of DVR2A^ΔNS1^ TCPs. Forty-height hours post-infection, cell lysates clarified by centrifugation were used for immunoprecipitation with HA-affinity agarose beads and eluates (IP) or whole cell lysates (Input) analyzed by western-blotting using antibodies specified on the right of each panel. Numbers on the left refer to molecular weight standards expressed in kDa; black arrowhead on the right indicates the ΔNS1 protein expressed by the DVR2A^ΔNS1^ genome. A representative experiment of four independent repetitions is shown. (**B**) *Interaction between NS1 and prM/E in DENV-2 wild-type virus-infected cells*. VeroE6 cells were mock infected or infected with DENV-2 at an MOI of 1. Forty-eight hours later clarified cell lysates were used for immunoprecipitation using a NS1-specific rabbit polyclonal antiserum or the corresponding pre-immune (PIS) antiserum and protein A-Sepharose beads. After extensive washing, eluted protein complexes were analyzed by western-blotting using polyclonal anti-NS1 and anti-prM or mouse monoclonal anti-E or anti-C specific antibodies as specified on the right of each panel. DENV proteins are indicated with arrowheads, asterisks refer to the immunoglobulin heavy chain.

Since NS1 secretion appeared functionally unlinked to infectious particle production, we next hypothesized that NS1 function(s) required for the late steps of the viral replication cycle might involve interactions between NS1 and the structural DENV proteins. To address this hypothesis we took advantage of our ΔNS1^TCP^ system using HA-tagged NS1 for trans-complementation. DVR2A^ΔNS1^ TCP stocks produced and titered in NS1^WT^ helper cells were used to infect NS1^HA^ target cells at an MOI of 1. Forty-eight hours later, cell lysates were subjected to HA-affinity capture using anti-HA agarose beads, and purified NS1 protein complexes or whole cell lysates were analyzed by western-blot using C-, prM-, E- and NS5-specific antibodies. Specificity of western-blot and immunoprecipitation analysis was monitored by including VeroE6_CTRL and VeroE6_NS1^WT^ cells, respectively. As shown in Fig 7A, upon infection of NS1 helper cells with ΔNS1^TCP^, comparable amounts of structural proteins accumulated in both NS1^WT^ and NS1^HA^ cells, confirming that DVR2A^ΔNS1^ replicates efficiently in both cell lines. Most interestingly, upon HA-immunoprecipitation, all three structural proteins (E, prM and C) specifically co-precipitated with NS1^HA^ arguing for an interaction between NS1 and DENV virions and possibly also subviral particles. In spite of comparable protein amounts in the cell lysates, no specific signal was detected in case of the non-tagged NS1^WT^ or for the NS5 protein, confirming specificity of the NS1^HA^-immunoprecipitation.

To corroborate the interaction between NS1 and the structural proteins, we performed analogous pull-down experiments using cells that had been infected with wild-type DENV. VeroE6 cells were mock-infected or infected with DENV-2 at an MOI of 1 and 48 hours later cell lysates were subjected to immunoprecipitation using a rabbit pre-immune serum (PIS) or an NS1-specific polyclonal antiserum (Fig 7B). Interestingly, also under these conditions E and prM were specifically co-immunoprecipitated with NS1 whereas C was not detected. The absence of C might be due to the overall lower efficiency of this immunocapture approach, to suboptimal conditions for antibody binding or to an altered ratio of subviral particles to infectious virions in the TCP system as compared to wild-type virus-infected cells (see discussion). Nevertheless, the interaction between E and NS1 was confirmed in a reciprocal approach by which the NS1 protein in DENV-2 infected cells could be specifically co-immunoprecipitated with envelope (S3 Fig). Altogether, these results support the notion that NS1 interacts with the viral envelope glycoproteins.

### Mapping of the site in NS1 required for interaction with the DENV envelope glycoproteins

**Fig 8:**
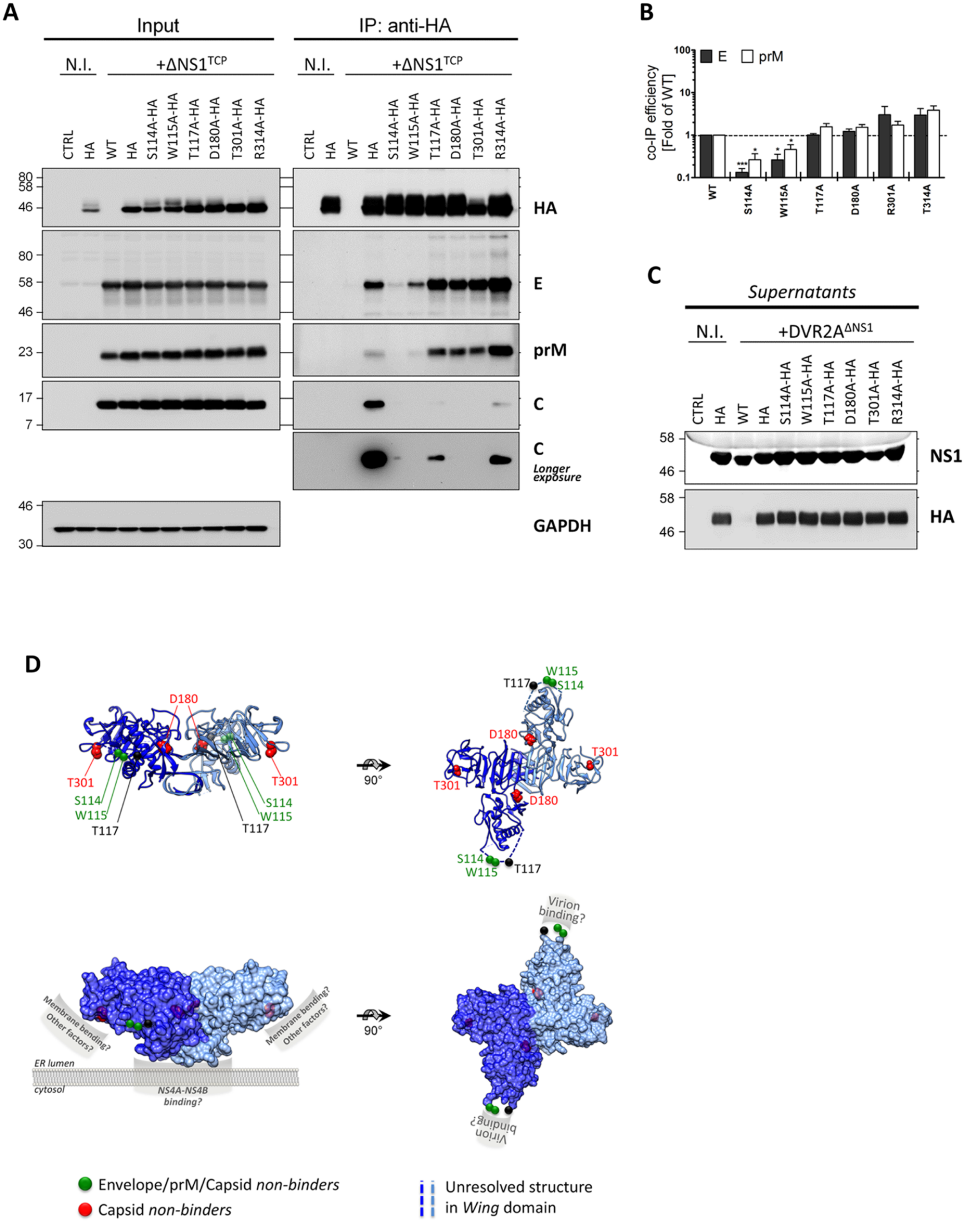
Exposed residues in the *Wing* and *β-ladder* domain of NS1 mediate interaction with DENV structural proteins. (**A**) *Pull-down of wild-type or mutant NS1 in the context of ΔNS1* ^*TCP*^ *infection*. Naïve VeroE6_NS1^WT^ (WT) or VeroE6 cells stably expressing different HA-tagged NS1 mutants were infected with 1 MOI of DVR2A^ΔNS1^. Two days post-infection, cell monolayers were lysed and subjected to HA pull-down as described in the legend to Fig 7B. Whole cell lysates (Input) or eluates (IP) were resolved on SDS-PAGE and analyzed by immunoblot using mono-specific antibodies given on the right of each panel. Numbers on the left refer to molecular weight standards expressed in kDa. (N.I., non-infected). (**B**) *Relative Envelope and prM IP efficiency*. Relative co-immunoprecipitation efficiency was calculated by densitometry normalizing the signals of Envelope or prM to immunoprecipitated NS1. Values represent mean and standard error of two to three independent experiments. (*** p<0.001; * p<0.05). (**C**) Cell culture supernatants from (A) were clarified by low-speed centrifugation, resolved on SDS-PAGE and analyzed by immunoblot using given antibodies. All the images are representative of 3 to 4 independent experiments. (**D**) *Localization of residues within the NS1 dimer involved in infectious particle production*. The DENV NS1 dimer structure (Protein Data Bank [PDB] accession no. 4O6B) is shown with each monomer represented in dark or light blue. Residues involved in infectious particle production are shown as *van der Waals* spheres. Mutations abrogating binding to E/prM/C or selectively preventing C binding are highlighted in green and red, respectively. *Upper panels*: ribbon representation of the NS1 dimer. *Lower panels*: putative functional NS1 domains involved in ER membrane binding, membrane bending and virion binding are highlighted. Note that these surfaces are exposed to the solvent and that the putative prM/E binding domain is located within the unresolved amino acid stretch of the *Wing* domain (aa 108–128; dashed lines).

Based on the results described above, we hypothesized that the selective defect in infectious particle production observed for some of the NS1 point mutants was due to an altered association with the envelope glycoproteins. To investigate this hypothesis, we analyzed the association of selected NS1 mutants with the structural proteins by co-immunoprecipitation in the DVR2A^ΔNS1^ TCP system, because it allowed highly efficient pull-down of NS1. We engineered stable VeroE6 cell lines expressing HA-tagged forms of the NS1 mutants S114A, W115A, T117A, D180A, T301A and R314A and infected these cell lines with ΔNS1^TCP^ at an MOI of 1. Samples harvested 48 h.p.i., together with positive and negative controls, were subjected to HA-specific pull-down and cell lysates, immunocomplexes or culture supernatants were analyzed as described above. All cell lines expressed HA-tagged NS1 variants and despite small variations in the expression levels rescued DVR2A^ΔNS1^ replication to similar extents as judged by the abundance of E, prM and C (Fig 8A) and the luciferase activity in the cell lysates (S5 Fig). Interestingly, analyses of NS1^HA^-immunocomplexes revealed marked differences in the interaction profiles of these mutants with the structural proteins. In case of NS1 mutants S114A and W115A, a significant reduction in E and prM co-precipitation, concomitant with a loss of the C-specific signal was observed, arguing for impaired association of these NS1 variants with DENV virions (Fig 8B; reduction of Envelope: ~7.5- and 3.8-fold, respectively). In contrast, mutants D180A and T301A exhibited a selective loss of co-precipitated C while retaining high prM and E interaction, suggesting that NS1 contributes to virus production in an additional manner that is independent from interaction with the envelope glycoproteins. Consistent with their higher competence in supporting infectious particle production, T117A and R314A were still able to bind all three structural proteins, although the C-specific signal was lower as compared to wild-type.

These results were further corroborated by co-localization analyses of HA-tagged NS1 mutants with the envelope glycoproteins revealing reduced co-localization in case of the two mutants that were impaired in virus production (S114A and W115A; S6 Fig), but no significant change in case of all the other mutants. Importantly, no major differences could be observed with respect to NS1 secretion into the culture supernatants (Fig 8C), strengthening the notion that NS1 secretion is functionally unlinked to its role in the production of infectious extracellular virus particles.

### NS1 co-localizes with assembled viral particles

**Fig 9:**
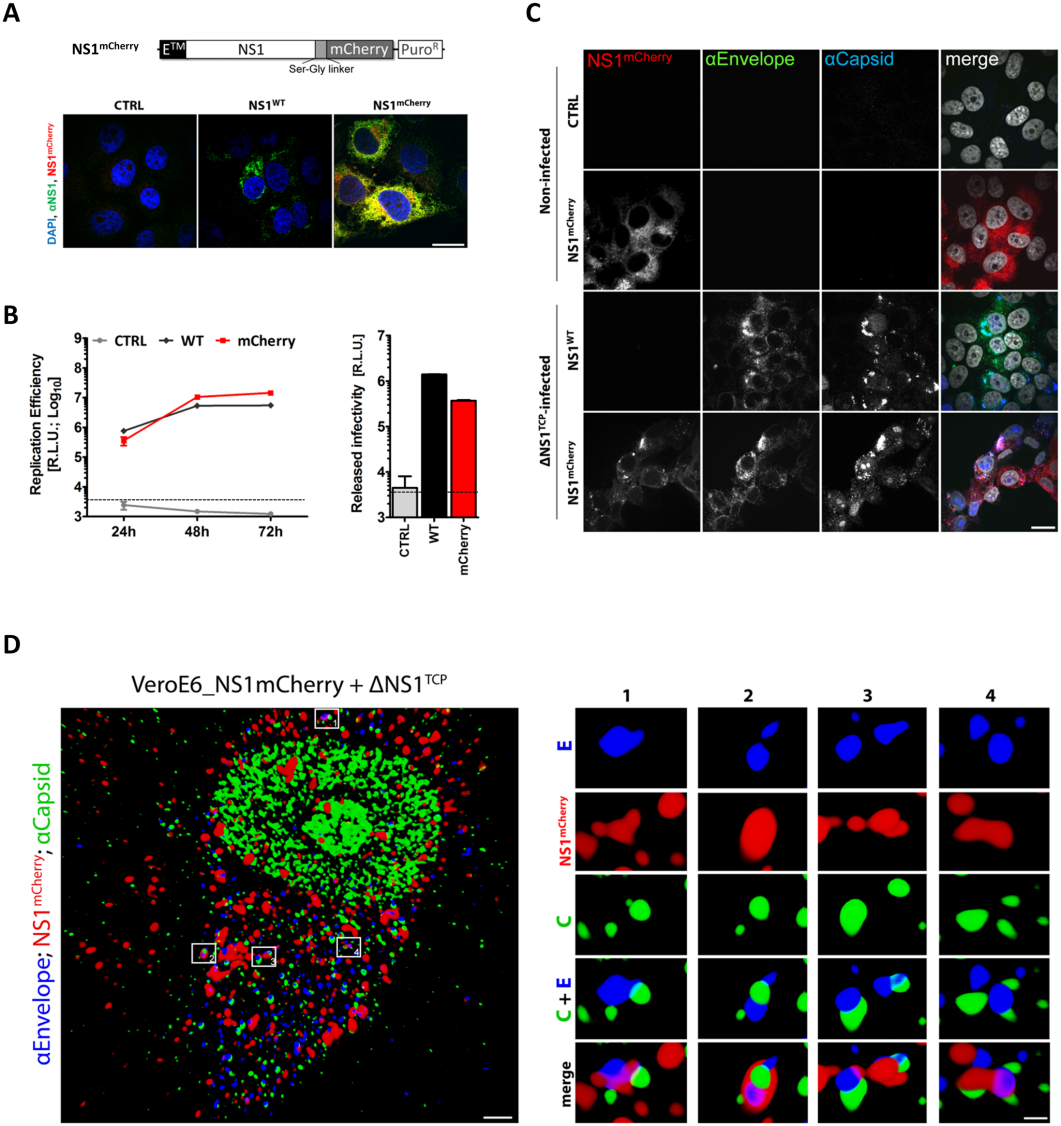
C-terminally mCherry-tagged NS1 supports DENV RNA replication and virus production and co-localizes with envelope and capsid upon ΔNS1^TCP^ infection. (**A**) *Sub-cellular distribution of NS1* ^*mCherry*^. Two days post-cell seeding, CTRL, NS1^WT^- or NS1^mCherry^-expressing cells were fixed, permeabilized and NS1 was detected with a rabbit polyclonal NS1-specific primary and AlexaFluor-488-conjugated secondary antibody. Cells were analyzed by confocal microscopy as described in materials and methods. The upper panel depicts a schematic representation of the NS1^mCherry^ fusion protein. (**B**) *Replication competence of ΔNS1* ^*TCP*^ *in VeroE6_NS1* ^*mCherry*^ *cells*. VeroE6 cells expressing no NS1 (CTRL) or NS1^WT^ or NS1^mCherry^ were infected with 1 MOI of ΔNS1^TCP^ and luciferase activity was measured in the lysates 24, 48 and 72 h post-infection. Released infectivity was assessed by inoculating NS1^WT^-expressing cells with culture media collected 72 h p.i. and measuring luciferase activity 48 h later. The dashed lines indicate the limit of detection of the assay. (**C-D**) *Co-localization and 3D reconstruction of NS1 with the structural proteins capsid and envelope*. (C) VeroE6 cells specified on the left, were mock-infected (Non-Infected) or infected with 1 MOI of ΔNS1^TCP^. Two-days later, cells were fixed, permeabilized and stained with E- and capsid-specific antibodies. Cells were analyzed by confocal microscopy. Scale bars represent 10 μm. (D) Deconvolved fluorescence image of VeroE6_NS1^mCherry^ cells infected with ΔNS1^TCP^ particles. VeroE6 cells stably expressing C-terminally tagged NS1^mCherry^ were infected with ΔNS1^TCP^ as described above. Two-days later, cells were fixed, permeabilized and stained with E- and capsid-specific antibodies. *Left panel*: representative image of an NS1^mCherry^- ΔNS1^TCP^-infected cell 48 h p.i. (scale bar represents 2 μm). Boxed areas are enlarged in the numbered panels on the right (scale bar represent 300 nm). Overview and enlargements show 3D reconstructed images created with the Imaris software as described in Materials and Methods.

To further investigate the interaction between NS1 and the structural proteins, we used immunofluorescence to visualize the sub-cellular colocalization of NS1 with the structural proteins. To allow detection of putative DENV assembly sites and/or assembled virus particles, we aimed to perform simultaneous immunostaining of capsid, envelope and NS1. Since we were limited by the availability of antibodies allowing for triple staining, we engineered a C-terminally mCherry-tagged NS1 variant (NS1^mCherry^) to be visualized without requirement for antibodies (Fig 9A). In the initial set of experiments, we confirmed correct NS1^mCherry^ expression and sub-cellular distribution. Importantly, mCherry-tagged NS1 supported efficient DENV RNA replication and infectious particle production upon infection with ΔNS1^TCP^ demonstrating full functionality of the fluorescently tagged NS1 (Fig 9B and 9C). Taking advantage of this approach, we analyzed infected cells by confocal microscopy and observed several discrete structures where Envelope, Capsid and NS1 colocalized (Fig 9D). Although the nature of these structures that might correspond to putative assembly sites or assembled virus particles is not clear, this colocalization provides additional evidence for a previously unreported association between NS1 and the structural DENV proteins.

**Fig 10:**
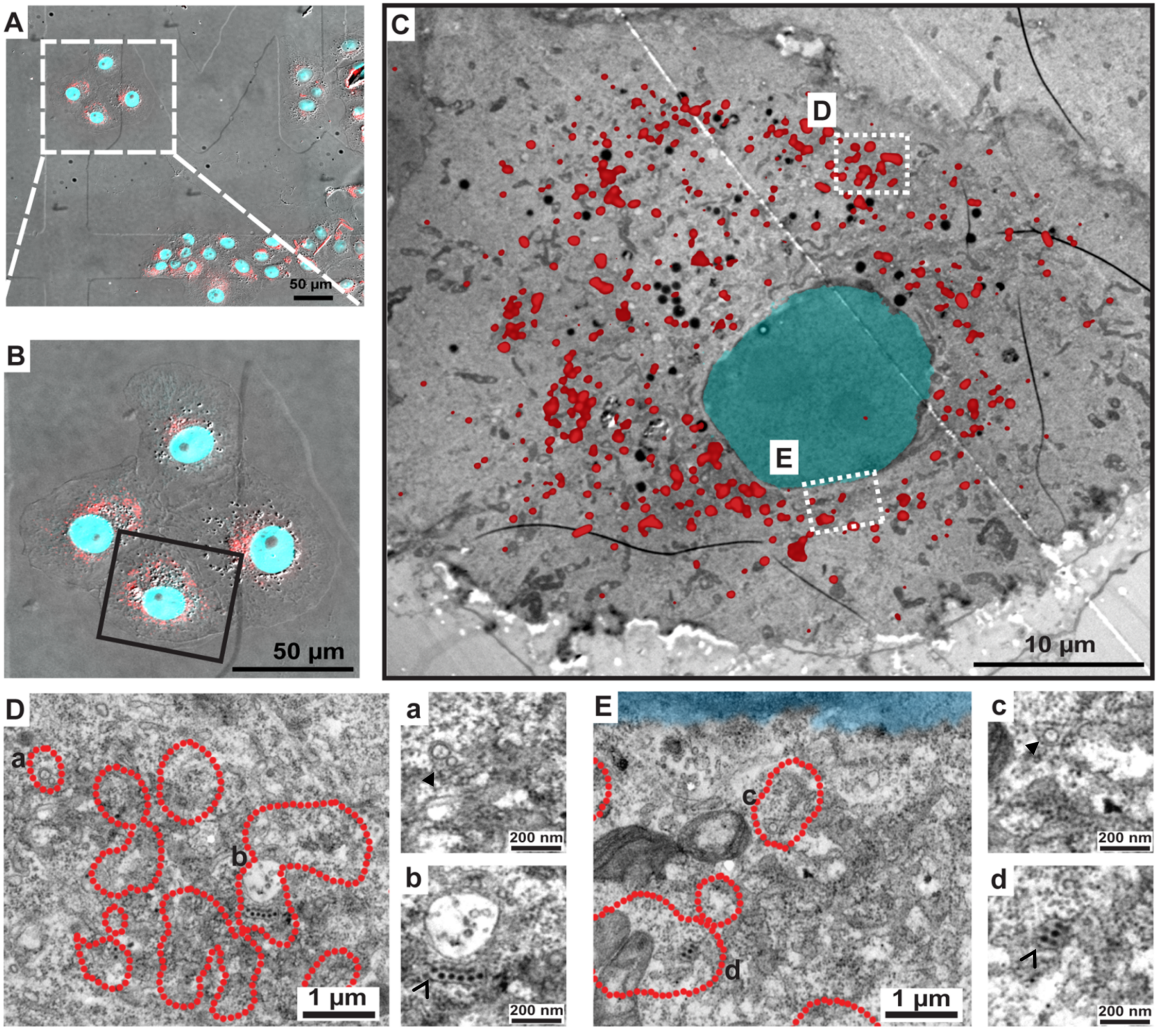
Correlative light-electron microscopy analysis identifies NS1 at sites of viral RNA replication and assembled virus particles. CLEM of VeroE6_NS1^mCherry^ cells infected with ΔNS1^TCP^. Cells were seeded on gridded coverslips and infected with 1 MOI of ΔNS1^TCP^. Forty-eight hours later, cells were fixed, analyzed by confocal microscopy and subsequently processed for EM as described in material and methods. (**A**) Merged image of bright field and mCherry-fluorescence image. Coordinates etched onto the surface of the gridded coverslip were used to record the position of the selected cells. (**B**) Enlarged fluorescence image of the cell of interest. (**C**) EM micrograph of the cell boxed in panel (B) overlapped with the fluorescence image. (**D-E**) Higher magnification images of the two different boxed regions in panel (C). Note that only the outer rim of each NS1^mCherry^ fluorescent area as in (C) is indicated with a red dotted line to facilitate visualization of membranous structures contained therein. Areas marked with lowercase letters in the left images are magnified in the corresponding right panels. (a-c) Full arrowheads indicate vesicle packets; (b-d) empty arrowheads indicate electron-dense virus particles.

To overcome the inherent limitations of fluorescence microscopy in allocating specific proteins to distinct subcellular structures and to elucidate the nature of these NS1-positive structures we performed correlative light-electron microscopy (CLEM) of VeroE6_NS1^mCherry^ cells infected with ΔNS1^TCP^ (Fig 10). MCherry-fluorescent structures were allocated by confocal microscopy of fixed cells grown on photo-etched gridded coverslips (Fig 10A and 10B), which were subsequently processed for EM (Fig 10C). After alignment of confocal and electron microscopy images we were able to identify NS1-enriched structures. As observed with NS1^HA^, NS1^mCherry^-infected cells also contained the characteristic DENV-induced membrane rearrangements. Indeed, we found that highly fluorescent mCherry-positive areas corresponded to ER, vesicle packets (VPs) and ER-associated bags containing arrays of DENV particles (Fig 9D and 9E). These results are consistent with our previous studies describing the association of NS1 with VPs as determined by immuno-EM [5]. Most importantly, the present data further support our assumption that NS1 associates with assembled virus particles (Fig 10Db and 10Ed), in agreement with our results from immunoprecipitation and confocal microscopy experiments.

## Discussion

Flavivirus NS1 has emerged as one of the most enigmatic proteins forming distinct intra- and extracellular complexes and contributing to pathogenesis as well as viral replication. While soluble and cell-surface–associated NS1 was shown to modulate complement activation pathways through interactions with host proteins, such as the regulatory protein factor H, complement factor C4 or clusterin [11–13,28] and to induce cross-reacting antibodies to human proteins [29–32], the role of NS1 in the viral replication cycle has so far been elusive. NS1 was initially thought to be involved in virus assembly or maturation, given its subcellular localization within the ER lumen and its secretion profile, largely mirroring that of the structural proteins prM and E [33,34]. However, this hypothesis was challenged by the co-localization of NS1 with dsRNA, a marker for RNA replication sites and by biochemical and genetic evidences supporting an essential role of NS1 in viral RNA replication [10,35–37]. Additionally, NS1 has been reported to interact with NS4B and/or NS4A, which are assumed to relay NS1 signals to other components of the viral replicase through their ER luminal segments.

In the present study, we used a combination of genetic, biochemical, and imaging approaches to investigate the functional role of intra- and extracellular NS1 in the DENV replication cycle. In addition to the previously reported mutations within the N-linked glycosylation sites [35,38] and the cysteine residues engaged in disulfide bonds [39], we identified 18 additional residues that completely or severely reduced DENV replication. Interestingly, most of these mutations clustered on residues located towards the proposed ER binding site (S1 Fig). Among these, a conserved tryptophan residue at amino acid position 8 (W8A) was found to be essential for RNA replication. This residue is located in close proximity to the previously reported di-amino acid motif (N10K11) within the *β-roll* domain of WNV NS1 that is also critical for efficient RNA replication [10,40]. These residues are located in an exposed region of the NS1 dimer suggested to face the ER membrane and possibly mediating the interaction with NS4B. Moreover, within the NS1 hexamer, these residues point towards the inner cavity of the barrel and might contribute to the association of NS1 with its lipid cargo (S1B Fig) [9]. While these results are consistent with the proposed way how the NS1 dimer associates with intracellular membranes and provide a detailed genetic map of NS1 residues essential for viral RNA replication, further studies are required to decipher the impact of these mutations on protein-protein interactions and induction of ultra-structural membrane rearrangements.

Most interestingly, our mutagenesis approach identified a group of NS1 mutants with selective defects in infectious particle production. These mutants (S114A, W115A, D180A and T301A) had only minor or negligible defects in NS1 protein stability and viral RNA replication as judged by their replication kinetics in the context of a sub-genomic replicon (Fig 4A), but released up to 100-fold lower amounts of infectious DENV particles than the wild-type (Figs 3A and 4B). Of note, amounts of intracellular infectivity were reduced ~5- to 10-fold, arguing that the NS1 mutations affected both assembly (as evidenced from intracellular virus titers) and release of infectious DENV particles (indicated by titer reduction in cell culture supernatants). Although we cannot precisely comment on the individual contribution of NS1 to virus assembly and particle release, the identification of an additional NS1 mutant (T117A) producing ~12-fold higher amounts of intracellular infectious virus strongly hints towards a primary role of NS1 in the assembly of infectious DENV particles.

**Fig 11:**
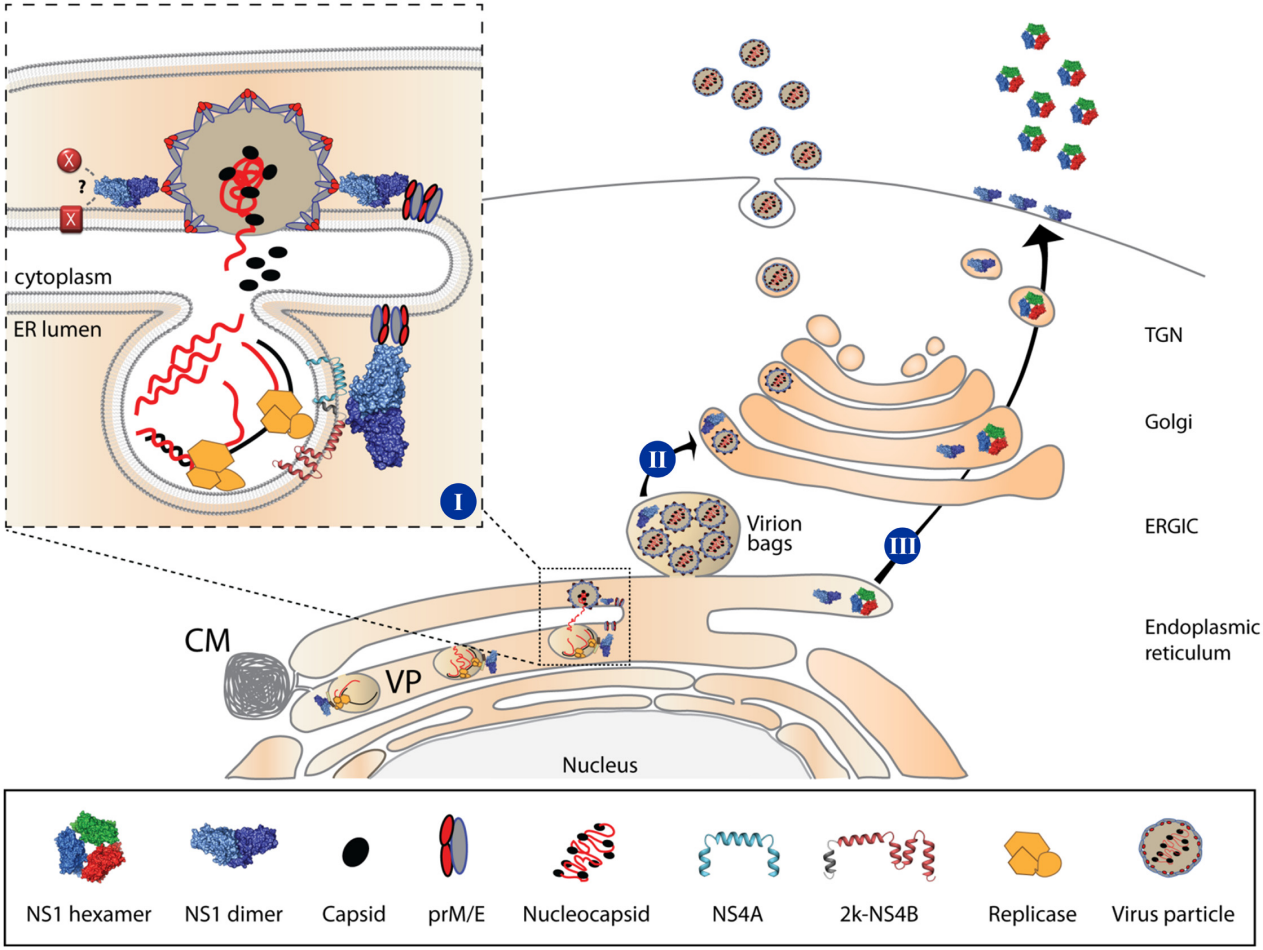
Proposed roles of NS1 in the DENV life cycle. [I] The NS1 dimer interacts with NS4A/NS4B through its *β-roll* domain, to assist early events in viral RNA replication. Additionally, NS1 interacts with the structural proteins prM/E through exposed residues in the *Wing* domain eventually to assist membrane bending and envelopment of nucleocapsids through exposed residues within the *β-ladder* domain, or via interaction with other viral or host factors within the endoplasmic reticulum. [II] Early vesicular traffic of assembled virus particles (i.e. from the ER to the ERGIC) might also be assisted by NS1; however, once in the Golgi, assembled virions can traffic to the plasma membrane and are secreted in the extracellular milieu independent from NS1. [III] NS1 is secreted as a hexameric lipoparticle independent from viral replication. Secreted NS1 is dispensable for viral RNA replication, virion assembly or particle release, since NS1^KDEL^ fully supports the complete replication cycle. However, secreted NS1 is required for counteracting immune responses. CM, Convoluted Membrane; VP, Vesicle Packet; ERGIC, ER-Golgi Intermediate Compartment; TGN, Trans-Golgi Network.

Based on previous studies reporting the ability of ectopically-expressed NS1 to trans-complement YFV or WNV NS1 deletion mutants [18,21], we established a DENV-based ΔNS1^TCP^ system to characterize NS1 functions. Taking advantage of this method, we probed the capacity of an intracellularly retained KDEL-tagged NS1 (NS1^KDEL^) to support production and release of viral particles and demonstrate that secretion of NS1 is dispensable for these functions. Of note, KDEL-tagged proteins are shuttling between the ER and the Golgi apparatus and thus, NS1^KDEL^ could still assist early vesicular traffic of viral particles, i.e. from virion budding sites (ER) to early secretory compartments (ERGIC) (Fig 11). Recently it has been reported that depletion of the KDEL receptor or a subset of class II Arf proteins reduces secretion of non-infectious sub-viral particles and that prM—KDEL receptor interaction plays a role in virus secretion, arguing that flavivirus release from infected cells is assisted by specific sorting mechanisms [41,42]. However, additional viral or host factors appear to be required to coordinate these processes, since knock-down of KDEL receptor or Arf4+5 expression reduced YFV and DENV1-3 secretion less than 10-fold and had no effect on DENV4 and WNV particle production. Further studies will be needed to elucidate the possible contribution of NS1 trafficking from the ER to early or intermediate secretory compartments for DENV particle release.

By using HA-tagged NS1 variants (NS1^HA^) and ΔNS1 virus-infected cells, we identified a previously unreported interaction between NS1 and the envelope glycoproteins E and prM. Although more than two decades ago E—NS1 complexes were identified in insect cells infected with a selected group of flaviviruses, the interaction was suggested to result from unspecific protein aggregation, lacking functional significance and not reproducible in DENV-2- and YFV-infected cells [26]. In contrast, several lines of evidence suggest that NS1 interaction with these structural proteins is specific and of importance for the production of infectious DENV particles: (i) the co-immunoprecipitation of NS1 with prM and E and, possibly indirectly, with C; (ii) the identification of triple-positive structures enriched in NS1, E and C by confocal microscopy; (iii) the detection of NS1 in compartments containing assembled virions by CLEM. Importantly, immunoprecipitation experiments using lysates of wild-type DENV-2-infected cells and an NS1-specific antibody confirmed the interaction of NS1 with both prM and E glycoproteins. Of note, while in this experimental set-up no specific signal could be detected for the capsid protein, it was well co-precipitated in the DVR2A^ΔNS1^ TCP system. This discrepancy might be due to the overall lower NS1 capture efficiency in case of wild-type virus-infected cells. Alternatively, it is possible that in wild-type virus-infected cells subviral particles (SVPs) are produced in higher abundance relative to virus particles, whereas in the DVR2A^ΔNS1^ TCP system production of virus particles might be favored relative to SVPs. Consistent with this assumption we observed a much faster and more efficient production of infectious virus particles in our TCP system than with wild-type virus-infected cells (Fig 6B). Assuming that NS1 can also interact with prM/E present on the surface of SVPs, in case of their excess production we would still observe coprecipitation of NS1 with the envelope glycoproteins, whereas C would no longer be co-precipitated. In contrast, when virus particles are produced in excess over SVPs, we would observe NS1 co-precipitation with prM/E and C, i.e. virions. This is consistent with the observed colocalization of NS1 with C and E (Fig 9) and the enrichment of NS1 in areas containing fully assembled DENV particles (Fig 10). Moreover, the conclusion is consistent with the membrane topology of E, prM and NS1 that are located in the lumen of the ER, while capsid resides on the cytoplasmic side of ER membranes (Fig 11). Since no tangible interactions between capsid and the glycoproteins were reported to date, the most likely explanation for the apparent NS1-C co-precipitation is an interaction between NS1 and assembled virions. While the precise mechanism remains to be determined, the specificity and functional relevance of these interactions was corroborated by the interaction profiles of NS1 mutants having defects in infectious particle production. Mutations affecting highly conserved residues of the flexible solvent-exposed loop in the NS1 *Wing* domain (S114A and W115A; Fig 8D) simultaneously abrogated C, prM and E association arguing that this NS1 loop is engaged in interaction with DENV virions. In contrast, mutations affecting residues in the *β-ladder* domain (D180A and T301A) preserved glycoprotein binding, but prevented association with capsid arguing for a second function of NS1 in the assembly of DENV particles that is independent from envelope protein interaction. While the exact mechanism remains to be established, it is tantalizing to speculate that NS1 might, directly or indirectly via these residues, interact with other viral or cellular factor(s) required for the formation of infectious DENV particles. Alternatively, NS1 might assist membrane budding or conformational changes in prM/E required for the envelopment of nucleocapsids. This hypothesis is supported by the localization of “capsid non-binder” mutants in the NS1 dimer structure. In fact, D180 resides at the intersection of the *Wing* and *β-ladder* domain and T301 points towards the ER membrane and is solvent exposed (Fig 8D). These mutations might affect the NS1 dimer fold, its affinity for membranes or its membrane-bending ability, while preserving prM/E-association through the distal tips of the *Wing* domain that points towards the ER lumen. Alternatively, these mutations might induce conformational constraints in prM/E complexes, reducing their plasticity and capability to envelope budding nucleocapsids, affect the recruitment of prM/E to assembly sites or only indirectly affect association with capsid, as a consequence of altered interactions with other viral [43,44] or host factors playing critical roles in virion morphogenesis. However, ultrastructural analysis of NS1 mutants failed to identify assembly intermediates, the only striking difference to the wild-type being a marked reduction in the overall amount of electron-dense particles found in proximity to or directly at the plasma membrane (S2 Fig). These results suggest that nucleocapsid formation and envelopment are coupled or that naked nucleocapsids have an extremely short half-life. Further experiments will be required to dissect the exact role of NS1 in the assembly process of infectious virus particles.

Assembly of flavivirus particles is a poorly understood process (reviewed in [45]). High-resolution imaging approaches have shed some light on the topological arrangement of viral RNA replication and virus particle assembly. The viral replicase machinery is assumed to reside in highly organized membranous structures, designated as vesicle packets (VPs). These structures are formed by ER invaginations containing pores that would allow the release of newly synthesized viral RNA to be used for packaging into virions [5,6]. While genetic evidence suggests the involvement of several non-structural proteins and host factors in flavivirus assembly [41,43,44,46–48], the underlying molecular mechanisms are not known. Besides the lack of tangible interactions between C and the prM/E complex, major limitations are posed by the difficulty to visualize assembly intermediates and the seemingly unspecific incorporation of genomic RNA into nucleocapsids [49,50]. Based on the results presented here, it is tempting to speculate that NS1 might assist virion morphogenesis via its lipid-remodeling activity [8,9], its affinity for membranes [9] and its ability to interact with both non-structural [10,15] and structural proteins. In this respect NS1 might provide essential lipids or recruit essential host factor required for the biogenesis of the replication complex, while coordinating the recruitment of E and prM to assembly sites juxtaposed to the viral replicase [5] (Fig 11).

In conclusion, the present study provides a comprehensive genetic map of NS1 determinants important for viral RNA replication and identifies a novel role of NS1 for the production of infectious DENV particles. We demonstrate that NS1 interacts with the envelope glycoproteins presumably on the surface of virions and these interactions are required for efficient production of infectious virus particles. Given its multiple roles in counteracting host defense, promoting RNA replication and enhancing production of virus particles, NS1 exemplifies the genetic economy of flaviviruses and emerges as attractive target for antiviral drugs.

## Materials and Methods

### Antibodies and sera

The mouse monoclonal antibody recognizing human GAPDH (sc-47724/0411) was purchased from Santa Cruz Biotechnology (Santa Cruz, CA). The mouse anti-Envelope monoclonal antibody (3H5-1) was purchased from ATCC. The mouse monoclonal antibody 6F3.1 reacting with the capsid protein was a kind gift of Dr. John G. Aaskov (Queensland University of Technology, Australia); rabbit polyclonal serum anti-capsid was a kind gift of Dr. Andrea Gamarnik (Fundación Instituto Leloir, Argentina). The rabbit polyclonal antibodies recognizing Envelope, NS1, NS5 and prM were previously described [5]. Rabbit anti-HA antibody (Ab9110) and mouse anti-NS1 antibody (ab41623) were purchased from Abcam. The mouse monoclonal anti-HA (H3663) and anti-Flag (F1804) antibodies, agarose anti-HA conjugated beads and secondary anti-mouse and anti-rabbit horse-radish peroxidase-conjugated antibodies were purchased from Sigma-Aldrich (Sigma-Aldrich, Saint Louis, MO).

### Cell culture

Huh7 [51], HeLa [52], VeroE6 (ATCC #CRL-1586), and BHK-21 (ATCC #CCL-10) cells were maintained in Dulbecco's modified Eagle medium (DMEM; Invitrogen, Karlsruhe, Germany) supplemented with 2 mM l-glutamine, nonessential amino acids, 100 U/ml penicillin, 100 μg/ml streptomycin and 10% fetal calf serum. VeroE6 stably expressing NS1^WT^ or tagged derivatives were cultured in the presence of 10 μg/ml of puromycin.

### Immunoblot analysis

Samples were denatured in 2x protein sample buffer (200 mM Tris [pH 8.8], 5 mM EDTA, 0.1% Bromophenolblue, 10% sucrose, 3% SDS, 1 mM DTT) and incubated for 5 min at 95°C. Proteins were separated by SDS-polyacrylamide gel electrophoresis (PAGE) and transferred onto polyvinylidene difluorid membranes by using a MINI-SDS-PAGE wet-blotting apparatus (Bio-Rad, Munich, Germany). Membranes were blocked with 5% non-fat dry milk in PBS/0.5% Tween-20 (PBST) and incubated with primary antibodies (capsid 1:50; GAPDH 1:1,000; HA 1:1,000; E 1:1,000; prM 1:500; NS1 1:500) by over-night incubation at 4°C or for 1 h at room temperature. After 3 washes with PBST, membranes were incubated with secondary horse radish peroxidase-conjugated antibodies, developed with the Western Lightning Plus-ECL reagent (Perkin Elmer; Waltham, MA) and bands were imaged using an Intas ChemoCam Imager 3.2 (Intas, Göttingen).

### Immunofluorescence analysis

VeroE6 helper cells expressing different forms of NS1 were infected with ΔNS1^TCP^ at an MOI of 1. Two days later, cells were fixed with 4% PFA for 10 min at room temperature, permeabilized with 0.5% (vol/vol) Triton X-100 in PBS and aspecific biding sites blocked with PBS containing 5% FBS for 30 min at RT.

For staining of NS1^mCherry^ cells, rabbit anti-NS1 (S3 Fig), or rabbit anti-C and mouse anti-Envelope antibodies (Fig 8) were used in combination with goat anti-mouse Alexa 647-conjugated and donkey anti-rabbit Alexa488-conjugated secondary antibodies. For staining of NS1^HA^ cells (wild-type and mutants) (S4 Fig), rabbit anti-HA and mouse anti-Envelope antibodies were used in combination with goat anti-mouse Alexa 568-conjugated and donkey anti-rabbit Alexa488-conjugated secondary antibodies. Nuclear DNA was stained with 4′,6-diamidino-2-phenylindole (DAPI) (Molecular Probes, Karlsruhe, Germany). Coverslips were mounted in Fluoromount-G mounting medium (Southern Biotechnology Associates, Birmingham, AL). For 3D visualization of NS1^mCherry^, E and C, samples were imaged with an Ultraview ERS spinning disk (PerkinElmer Life Sciences) on a Nikon TE2000-E inverted confocal microscope using a Plan-Apochromat VC 100× objective (numeric aperture [NA], 1.4). Optical sections of 0.13 μm were acquired separately for each channel. Z-stacks were deconvolved with a theoretical point-spread function, and chromatic shifts between green and far-red dyes were corrected using Autoquant X3 software. 3D reconstructed images were created using the Imaris 8 software package. For colocalization analyses of HA-tagged NS1 mutants and envelope fluorescence signals, Pearson's correlation coefficient was calculated on single plane images, by using the integrated function in Fiji (ImageJ).

### Infectivity assays

For determination of virus titers by limiting dilution assay, Huh7 target cells were seeded into 96-well plates (10^4^ cells/well) the day before infection. Cells were inoculated with serial dilutions of virus-containing supernatants that had been filtered through a 0.45-μm-pore-size filter. Infected cells were detected by immune staining of the E protein using the mouse anti-E antibody (3H5-1; diluted 1:500) and secondary horseradish peroxidase-conjugated antibody (1:200). Virus titers (expressed as 50% tissue culture infective dose [TCID_50_]/ml) were calculated as previously reported [53]. For determination of virus titers by Focus forming unit (FFU) assay, VeroE6 or VeroE6_NS1^WT^ cells (2x10^5^ cells/well) seeded into 24-well plates, were infected with serial dilutions of 0.45-μm-filtered supernatants and incubated in the presence of 0.8% methylcellulose for 5 days. Monolayers were rinsed twice in PBS, fixed with 5% PFA and permeabilized with 0.2% (v/v) TritonX-100 in PBS for 15 min. Infected foci were detected by immune staining of the E protein using the mouse anti-E antibody (3H5-1; diluted 1:1,000 in PBS) and secondary horseradish peroxidase-conjugated antibody (1:200). Alternatively, virus titers were determined by standard plaque assay (PFU) on target VeroE6 cells as previously described [54]. To determine intracellular infectivity titers, transfected cells were disrupted by several freeze–thaw cycles as described earlier [55]. In brief, transfected Huh7 cells were extensively washed with PBS, scraped off the plate into PBS and centrifuged for 5 min at 700 × g. Cell pellets were resuspended in complete DMEM (containing 15 mM HEPES, pH 7.2–7.5) and subjected to three cycles of freezing and thawing by using liquid nitrogen and a thermo block set to 37°C. Cell debris was removed by centrifugation at 20,000 × g for 10 min at 4°C. Virus-containing culture supernatants from transfected cells were treated in the same way and infectivity was determined in parallel by limiting dilution assay as described above.

### Co-immunoprecipitation assay

VeroE6 cells were seeded into 15-cm^2^ dishes (7.5x10^6^ cells/dish). Twenty-four hours later, cell monolayers were either mock-infected or infected with DENV-2 (MOI = 1). Forty-eight hours later, cell monolayers were scraped into 1 ml lysis buffer (50 mM Tris-HCl [pH 8.0], 0.5% NP-40, 150 mM NaCl and protease inhibitor cocktail (cOmplete, Roche)). After 30 min incubation on ice, cell debris was removed by 15 min centrifugation at 13,800xg and 400 μl of clarified cell lysate was incubated with rabbit pre-immune serum (PIS) or rabbit anti-NS1 antiserum for 6 hours at 4°C in a head-to-head shaker. Samples were incubated with 40 μl of protein A beads slurry (Sigma Aldrich, St. Louis, USA) for 1 hour at 4°C, washed three times with 1 ml of lysis buffer and protein A-bound complexes were transferred into a fresh tube. After a final wash with 1 ml of lysis buffer for 15 min at 4°C, protein complexes were eluted at room-temperature by two consecutive steps with 75 μl of 0.1 M glycine [pH 2.5] for 5 min. Collected supernatants were immediately neutralized by adding 7.5 μl 1 M Tris-HCl [pH 8] and denatured for 5 min at 95°C in the presence of 33 μl of 6X SDS sample buffer. Alternatively, mouse anti-HA or mouse anti-E antibodies were used in combination with protein G beads slurry (Sigma Aldrich, St. Louis, USA) and the immunoprecipitation was carried exactly as described above. For ΔNS1^TCP^ experiments, VeroE6 cells stably expressing an empty pWPI vector (CTRL), or wild-type NS1 (NS1^WT^), or HA-tagged NS1 (NS1^HA^) or derivatives thereof were seeded into 10-cm^2^ dishes (3x10^6^ cells/dish). Twenty-four hours later, cell monolayers were infected with DVR2A^ΔNS1^ (MOI = 1) for 4 h at 37°C. Forty-eight hours post-infection, cell monolayers were scraped into 1 ml lysis buffer (50 mM Tris-HCl [pH 8.0], 0.5% NP-40, 150 mM NaCl and protease inhibitor cocktail (cOmplete, Roche) as recommended by the manufacturer). After 30 min incubation on ice, cell debris was removed by 15 min centrifugation at 13,800xg. For HA-specific affinity capture, samples were incubated with HA-specific agarose beads (Sigma-Aldrich, St.Louis, USA) for 5 h by continuously inverting the tubes at 4°C. Beads were washed three times for 20 min with large volumes of lysis buffer at 4°C and samples were eluted at room-temperature in two consecutive steps with 3% SDS in PBS for 5 min and PBS for 5 min, respectively. The two eluates were pooled and precipitated over-night at -20°C with 4 volumes of ice-cold acetone. Samples were centrifuged for 30 min at 20,000xg, air-dried, resuspended in 2x SDS sample buffer and boiled for 5 min at 95°C. Alternatively, 4 h before ΔNS1^TCP^ infection, VeroE6 cells stably expressing wild-type NS1 (NS1^WT^) or HA-tagged NS1 (NS1^HA^) were transfected with 10 μg of pcDNA 3.1(+) empty vector or pCMV_NS4B-FLAG (encoding a C-terminally Flag-tagged NS4B protein of Hepatitis C virus) by using the TransIT-LT1 transfection reagent (MirusBio LLC, Madison, WI, USA) as recommended by the manufacturer. Infection and HA-specific immunoprecipitation were carried out exactly as described above. Eluted proteins were further analyzed by western blot as specified in the results section. For analysis of secreted NS1, supernatants were clarified through 0.45 μm filters and incubated with 40 μl mouse anti-HA slurry beads over-night at 4°C, in a head-to-head shaker. Immunoprecipitates were washed three times with lysis buffer and eluted as described above. Alternatively cell culture supernatants containing NS1 were used undiluted for SDS-PAGE.

### Electroporation of DV, DVR2A and sgDVR2A *in vitro* transcripts into mammalian cells

*In vitro* transcripts were generated as previously described [56]. For RNA transfection, single-cell suspensions were prepared by trypsinization, washed with PBS, and resuspended at a concentration of 1x10^7^ cells (Huh7) or 1.5x10^7^ cells (VeroE6) per ml in Cytomix, supplemented with 2 mM ATP and 5 mM glutathione. Five to 10 μg of subgenomic or genomic *in vitro* transcript was mixed with 400 μl of the cell suspension and transfected by electroporation using a Gene Pulser system (Bio-Rad) and a cuvette with a gap width of 0.4 cm (Bio-Rad) at 975 μF and 270 V. Cells were immediately diluted into 20 ml of DMEM cplt and seeded in the appropriate format (1ml/well in 24-well plates; 2 ml/well in 12-well plates; 15 ml/dish in 15 cm-diameter dishes).

### Production of lentiviruses

Human immunodeficiency virus (HIV)-based particles that were pseudotyped with the vesicular stomatitis virus glycoprotein (VSV-G) were generated by transfection of 293T cells as described previously [53]. For production of transducing lentiviral particles, 293T cells were co-transfected with a transfer vector encoding the gene of interest and a puromycin resistance gene (pWPI_Puro), the HIV-1 packaging plasmid (pCMV) and a VSV-G expression vector (pMD.G) (ratio 3:3:1). Cells were transfected using the CalPhos mammalian transfection kit as recommended by the manufacturer (Becton Dickinson). After 48 and 72 h, supernatants were harvested, clarified through 0.45 μm pore size filters, pooled and stored in aliquots at -20°C until use. Titers of lentiviral particles were estimated by colony-forming unit (CFU) assay using HeLa cells and serial dilutions of each lentiviral stock. Inoculated cells were subjected to selection using the appropriate antibiotic for 5–7 days and surviving cell colonies were stained for 15min with a 1% crystal violet solution. Colonies were counted under a bright-field inverted microscope and lentivirus titers were calculated as CFU/ml.

### Transient replication assay

Huh7 or VeroE6 cells transfected with full-length or subgenomic DVsR2A in vitro transcripts were seeded as specified in the results section (typically 12- or 24-wells plates). Replication was determined by measuring luciferase activity in cell lysates 4, 24, 48 and 72 h after transfection. For determination of luciferase activity, cells were washed once with PBS and lysed by adding 200 μl of luciferase lysis buffer as previously described [57]. Cells were frozen immediately at −70°C and after thawing, lysates were resuspended by gentle pipetting. For each well 20 μl lysate, mixed with 400 μl assay buffer (25 mM glycylglycine, 15 mM MgSO_4_, 4 mM EGTA, 1 mM DTT, 2 mM ATP, 15 mM K_2_PO_4_ [pH 7.8], 1.42 μM coelenterazine H), were measured for 10 sec in a tube luminometer (Lumat LB9507, Berthold, Freiburg, Germany). In some cases (24-well plates, 100 μl lysis buffer per well) a plate luminometer was used (Mithras LB940, Berthold, Freiburg, Germany). Each well was measured in duplicate. To determine the amount of infectious virus particles released into culture supernatants 72 h after electroporation, naïve VeroE6 cells were inoculated with culture supernatants of transfected cells and 48 h later luciferase activity was determined. Kinetics of virus replication were calculated by normalizing the relative light units (RLU) measured at a given time point to the respective 4 h value.

### Plasmids

The plasmid containing a synthetic version of the full-length DENV-2 strain 16681 (pFK-DVs) and subgenomic constructs derived therefrom without or with luciferase reporter were previously described [56]. For NS1-targeted site-directed mutagenesis external primers NS1_BamHI_f (5’-CTG GGA TTT TGG ATC CTT GGG AGG AG-3’) and NS1_KasI_r (5’-TCC GTC ATA GTG GCG CCT ACC ATA AC-3’) were used in combination with mutagenic forward and reverse primers (the full list of primers is available upon request). Amplicons containing the desired point mutation in the NS1 coding region were inserted into the full-length DVsR2A constructs via the *BamHI-KasI* restriction sites. Full-length non-reporter constructs containing selected NS1 mutations were generated by insertion of a DNA fragment that was excised *via BamHI* and *KasI* from pFK-DVsR2A into pFK-DVs; subgenomic luciferase reporter constructs were generated by DNA fragment exchange using *MluI* and *KasI* and insertion into pFK-sgDVsR2A. The pCMV_NS4B-FLAG expressing C-terminally Flag-tagged NS4B protein of Hepatitis C virus was previously described [58]. The pWPIpuro-based NS1 constructs used for production of lentiviral vectors were generated by PCR-based amplification of the NS1 encoding sequence plus the last 24 codons of the envelope coding region, by using full-length genomic constructs as template and the following primers: pWPIpuro_BamHI_NS1_HA_frw (5’-GCT GGG ATC C ACC ATG AGC ACC TCA CTG TCT GTG ACA CTA GTA TTG GTG-3’) and pWPIpuro_NS1_Stop_NheI_rev (5’-AGA TAG CTA GCC TAA GCT GTG ACC AAG GAG TTG ACC AAA TTC-3’). Amplicons were inserted into pWPI-Puro via *BamHI* and *SpeI* restriction sites. For variants encoding the C-terminal HA epitope or the KDEL ER retrieval sequence, the primer pWPIpuro_BamHI_NS1_HA_frw was used in combination with NheI_NS1-HA_stop_rev (5’-ATA GCT AGC CTA AGC GTA ATC TGG AAC ATC GTA TGG GTA TGA TCC AGC TGT GAC CAA GGA GTT GAC CAA ATT CTC TTC TTT CT-3’) or pWPIpuro_NS1_Stop_NheI_KDEL_rev (5’-AGA TAG CTA GCC TAT AGC TCG TCC TTA GCT GTG ACC AAG GAG TTG ACC-3’), respectively.

The pWPIpuro-based NS1^mCherry^ expression construct was generated by amplifying the mCherry sequence contained in pFKI389neoNS3-3′δg_JFH-1_NS5A-aa2359_mCherry [59] with primers pWPIpuro_SpeI_mCherry_frw (5´-TCA ACT CCT TGG TCA CAG CTA CCG GTG GAT CGA TGG TGA GCA AGG GCG AGG A-3´) and pWPIpuro_SpeI_mCherry_rev (5´-AAA ACT AGT CTA CTT GTA CAG CTC GTC CAT GC-3´) and the NS1 coding sequence contained in pWPIpuro_NS1 with primers pWPIpuro_SpeI_NS1_frw (5´-GAC ACT AGT ATT GGT GGG AAT TGT GAC AC-3´) and pWPIpuro_SpeI_NS1_rev (5´-TCC TCG CCC TTG CTC ACC ATC GAT CCA CCG GTA GCT GTG ACC AAG GAG TTG A-3´). The two PCR fragments were used to generate an intermediate amplicon using primers pWPIpuro_SpeI_NS1_frw and pWPIpuro_SpeI_mCherry_rev, which was inserted into pWPIpuro via *SpeI*. Finally, the envelope leader peptide sequence was excised from pWPIpuro_NS1 *wild-type* and inserted upstream of the NS1^mCherry^ sequence via *BamHI-MluI*.

The ΔNS1 full-length DVsR2A genome used for trans-complementation studies in NS1 helper cell lines, was created by insertion of a 97 codon in-frame deletion into the NS1 open reading frame using NS1_BamHI_f and NS1_KasI_rev as external primers and NS1_156_frw (5’-GAA TTC GTT GGA AGT TGA ACA CAA CTA TAG ACC AGG CTA-3’) and NS1_156_rev (5’-TAG CCT GGT CTA TAG TTG TGT TCA ACT TCC AAC GAA TTC-3’) as internal primers. Thus, in the final construct, the first 156 codons and the last 99 codons of NS1 were retained, respectively.

### Sequence alignments and molecular graphics

Sequence alignment of NS1 open-reading frames were performed using the ClustalW algorithm available in the JalView Desktop software and a ClustalW scoring algorithm, with the following isolates (UniprotKB/Swiss-Prot accession numbers are given): DV-1 (Brazil/97-11/1997) *P27909;* DV-2 (Thailand/16681-PDK53) *P29991*; DV-3 (Martinique/1243/1999) *Q6YMS3*; DV-4 (Thailand/0348/1991) *Q2YHF0*; West Nile virus *P06935*; Yellow Fever (Ivory Coast/1999) *Q6J3P1*; Japanese Encephalitis virus (SA-14) *P27395*; Kunjin virus (MRM61C) *P14335*; St. Louis Encephalitis virus (MS1-7) *P09732*. Molecular graphics were performed with the UCSF Chimera package developed by the Resource for Biocomputing, Visualization, and Informatics at the University of California, San Francisco [60] on the DENV-2 NS1 crystal structure (Protein Data Bank [PDB] accession no. 4O6B).

### Analysis of infected cells by transmission electron microscopy

VeroE6-based helper cell lines expressing different NS1^HA^ mutants were seeded onto glass coverslips (5x10^4^ cells/well) and 16 h later, infected with 1 MOI of DVR2A^ΔNS1^. After a 48 h incubation period, cells were fixed and prepared for transmission electron microscopy as described previously [61]. For correlative light-electron microscopy, VeroE6-based helper cell lines expressing NS1^mCherry^ were seeded into glass-bottom culture dishes containing photo-etched gridded coverslips (MatTek Corporation, Ashland, MA) and infected with 1 MOI of DVR2A^ΔNS1^. After 48 hours, cells were fixed with 4% PFA and 0.2% glutaraldehyde in PBS for 30 min at room temperature, washed three times with PBS, stained with DAPI and analyzed by fluorescence microscopy to acquire optical sections of 0.13 μm as described above. NS1^mCherry^-positive cells were imaged and their position on the gridded coverslip was recorded. Cells were then processed for analysis as described previously [61]. The DAPI signal was used for correlation purposes and images were adapted by using the Image J (version 1.46r) and Adobe Photoshop (version 12.1.1) software packages.

### Statistical analyses

Statistical analyses were performed by applying the two-tailed, unpaired Student’s t-test available within the GraphPad Prism (ver. 5.0) software.

## Supporting Information

S1 Fig Localization of amino acid residues involved in viral RNA replication within the 3D crystal structure of NS1. (**A**) *Localization of essential amino acid residues within the NS1 dimer involved in viral RNA replication*. The DENV NS1 dimer structure (Protein Data Bank [PDB] accession no. 4O6B) is shown with each monomer represented in dark or light blue. Residues involved in viral RNA replication are shown as *van der Waals* spheres. Residues identified in this study are highlighted in magenta, while the previously identified di-amino acid motif (N10K11; [10]) is indicated in green. (**B**) *Localization of residues in β-roll and β-ladder domains involved in viral RNA replication within the NS1 hexamer*. The NS1 hexameric structure is shown in *ribbon* with dimers represented in blue, red and green. Amino acid residues within the *β-roll* domain and the greasy finger of the *β-ladder domain* involved in viral RNA replication are given in colored *van der Waals* spheres. Note that they face the inner cavity of the NS1 hexamer. Shown in green is the N10K11 motif, while in magenta residues W8, W158 and G161 identified in the present study.
(TIF)

S2 Fig Ultrastructural analyses of NS1 assembly/release mutants in the context of ΔNS1^TCP^ infection and HA-tagged stable cell lines. VeroE6 helper cells stably expressing different NS1^HA^ mutants specified on the left of each panel or VeroE6_NS1^HA^ wild-type-expressing cells (NS1-HA) were either mock-infected (non-infected) or infected with 1 MOI of DVR2A^ΔNS1^ TCPs. Forty-eight hours post-infection, cells were fixed and analyzed by transmission electron microscopy as described in materials and methods. The areas boxed in the left panels are shown at higher magnification on the right. Black and white scale bars represent 1 μm and 200 nm, respectively.
(TIF)

S3 Fig Association of NS1 with PrM/E in DENV-2 infected cells. VeroE6 cells were mock infected or infected with DENV-2 at an MOI of 1. Forty-eight hours later clarified cell lysates were used for immunoprecipitation using anti-E or anti-HA mouse monoclonal antibodies and protein G-Sepharose beads. After extensive washing, eluted protein complexes were analyzed by western-blotting using polyclonal anti-NS1 and anti-E specific antibodies as specified on the right of each panel. Arrowheads indicate DENV proteins; asterisks refer to the immunoglobulin heavy chain.
(TIF)

S4 Fig DENV NS1 does not interact with an unrelated ER-resident transmembrane protein. VeroE6_NS1^WT^ (WT) or VeroE6_NS1^HA^ (HA) helper cells were transfected with pcDNA3.1 or Flag-tagged NS4B of the Hepatitis C virus (HCV-NS4B^FLAG^). Four hours later, cell monolayers were washed with PBS and infected with DVR2A^ΔNS1^ TCPs (MOI = 1). Forty-eight hours post-infection, cell lysates clarified by centrifugation were used for immunoprecipitation with HA-affinity agarose beads and eluates (IP) or whole cell lysates (Input) analyzed by western-blotting using antibodies specified on the right of each panel. Numbers on the left refer to molecular weight standards given in kDa.
(TIF)

S5 Fig Replication of NS1^HA^ mutants upon infection with DVR2A^ΔNS1^ TCPs. Naïve VeroE6 cells stably expressing *wild-type* NS1^HA^ (HA) or different HA-tagged NS1 mutants were infected with 1 MOI of DVR2A^ΔNS1^ TCPs. Forty-eight hours later luciferase activity was measured in the lysates to determine replication efficiency.
(TIF)

S6 Fig Subcellular localization of NS1 mutants in ΔNS1 virus infected cells. (**A**) Subconfluent VeroE6_NS1^HA^ cells (NS1^HA^) or VeroE6 helper cells stably expressing different NS1^HA^ mutants specified on the left of each panel, were infected with 1 MOI of DVR2A^ΔNS1^ or mock infected (non-inf). Forty-eight hours later, cells were fixed and immunostained with rabbit HA- and mouse Envelope-specific antibodies. Scale bar represents 10 μm. (**B**) Co-localization of NS1 and E in the experiments shown in panel A was assessed by using the coloc2 plug-in within the Fiji (ImageJ) software package. Values represent mean and standard errors of Pearson’s correlation coefficients from at least 25 individual cells per condition. ***, P < 0.001.
(TIF)
