# Supplementary figures and images for: Dengue Virus Non-structural Protein 1 Modulates Infectious Particle Production via Interaction with the Structural Proteins
Source: PLoS Pathog. 2015 Nov 12;11(11):e1005277. doi: 10.1371/journal.ppat.1005277 (PMC4643051; doi:10.1371/journal.ppat.1005277)

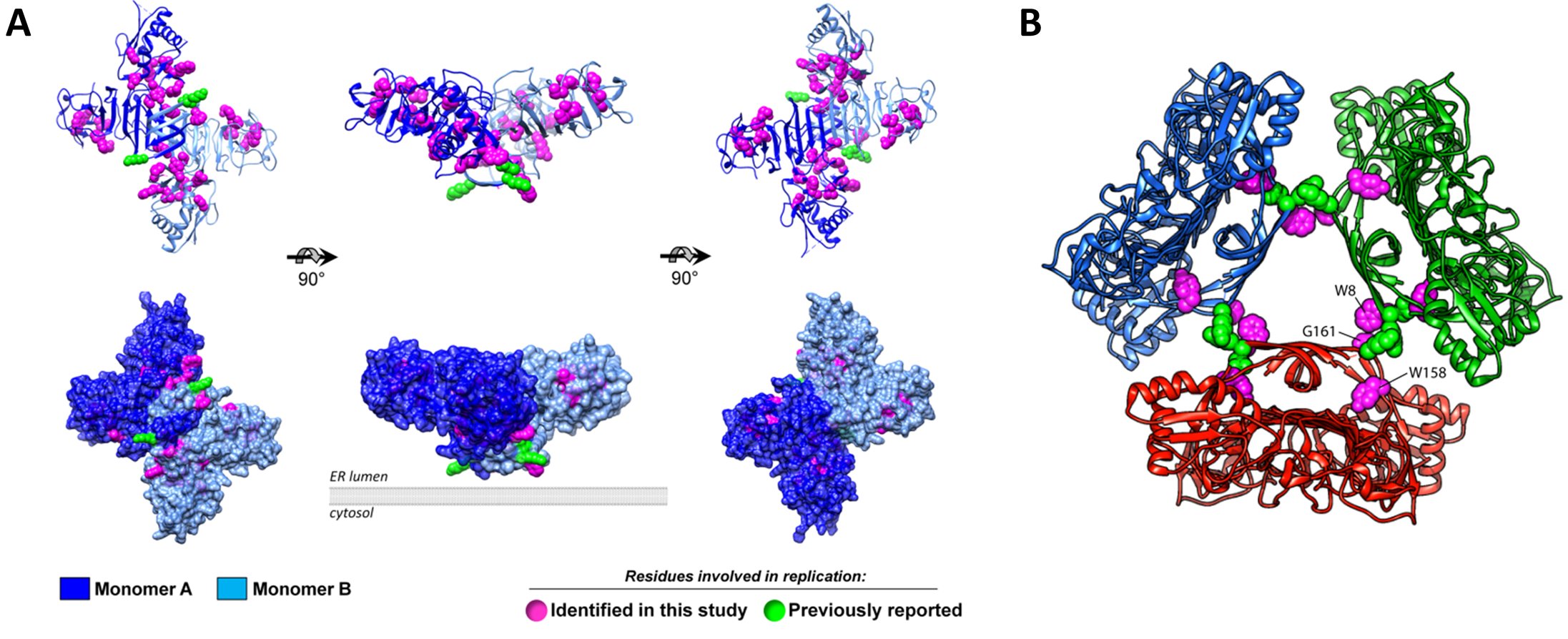

Supplement: S1 Fig — (A) Localization of essential amino acid residues within the NS1 dimer involved in viral RNA replication. The DENV NS1 dimer structure (Protein Data Bank [PDB] accession no. 4O6B) is shown with each monomer represented in dark or light blue. Residues involved in viral RNA replication are shown as van der Waals spheres. Residues identified in this study are highlighted in magenta, while the previously identified di-amino acid motif (N10K11; [10]) is indicated in green. (B) Localization of residues in β-roll and β-ladder domains involved in viral RNA replication within the NS1 hexamer. The NS1 hexameric structure is shown in ribbon with dimers represented in blue, red and green. Amino acid residues within the β-roll domain and the greasy finger of the β-ladder domain involved in viral RNA replication are given in colored van der Waals spheres. Note that they face the inner cavity of the NS1 hexamer. Shown in green is the N10K11 motif, while in magenta residues W8, W158 and G161 identified in the present study. (TIF) [file ppat.1005277.s001.tif]

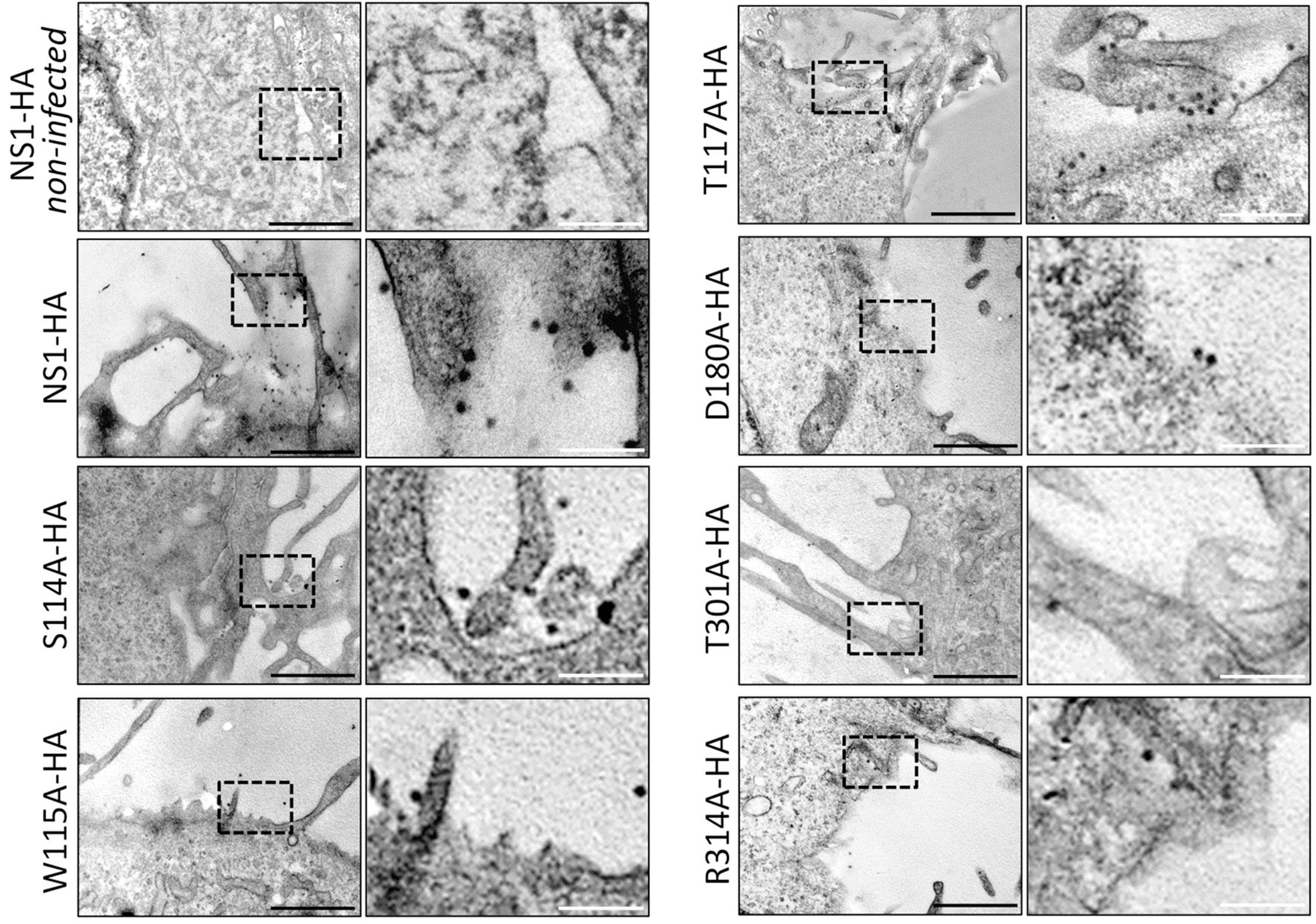

Supplement: S2 Fig — VeroE6 helper cells stably expressing different NS1HA mutants specified on the left of each panel or VeroE6_NS1HA wild-type-expressing cells (NS1-HA) were either mock-infected (non-infected) or infected with 1 MOI of DVR2AΔNS1 TCPs. Forty-eight hours post-infection, cells were fixed and analyzed by transmission electron microscopy as described in materials and methods. The areas boxed in the left panels are shown at higher magnification on the right. Black and white scale bars represent 1 μm and 200 nm, respectively. (TIF) [file ppat.1005277.s002.tif]

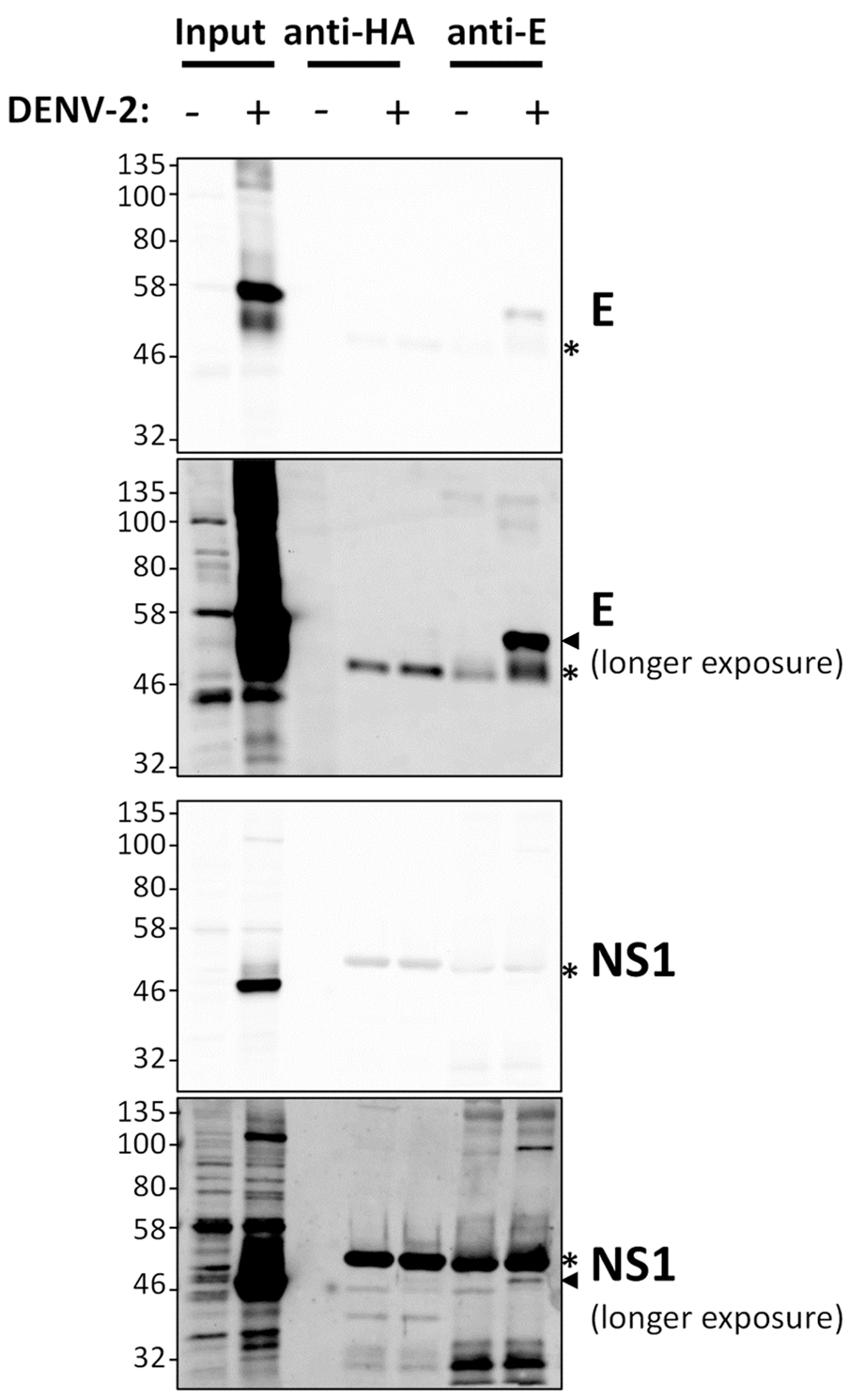

Supplement: S3 Fig — VeroE6 cells were mock infected or infected with DENV-2 at an MOI of 1. Forty-eight hours later clarified cell lysates were used for immunoprecipitation using anti-E or anti-HA mouse monoclonal antibodies and protein G-Sepharose beads. After extensive washing, eluted protein complexes were analyzed by western-blotting using polyclonal anti-NS1 and anti-E specific antibodies as specified on the right of each panel. Arrowheads indicate DENV proteins; asterisks refer to the immunoglobulin heavy chain. (TIF) [file ppat.1005277.s003.tif]

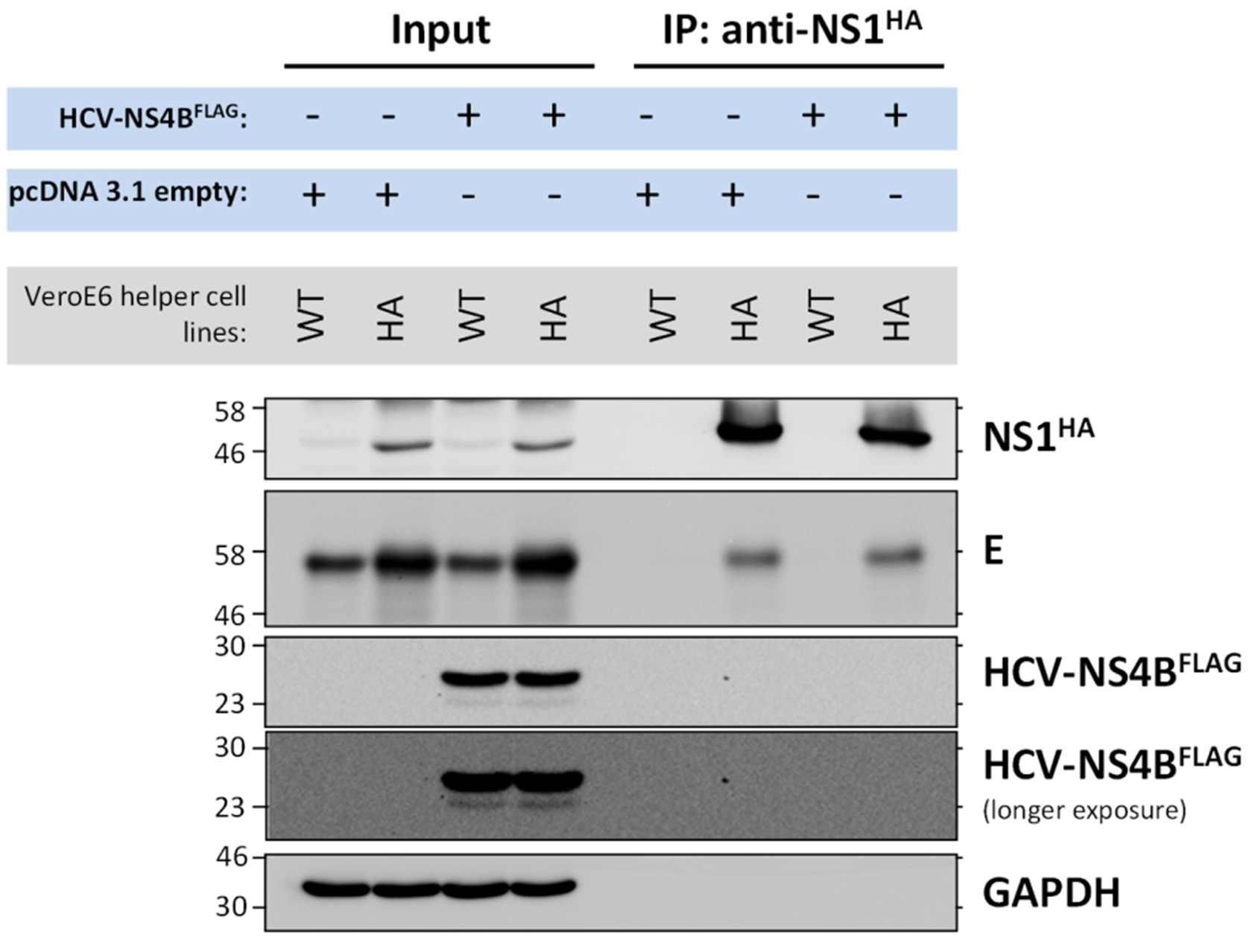

Supplement: S4 Fig — VeroE6_NS1WT (WT) or VeroE6_NS1HA (HA) helper cells were transfected with pcDNA3.1 or Flag-tagged NS4B of the Hepatitis C virus (HCV-NS4BFLAG). Four hours later, cell monolayers were washed with PBS and infected with DVR2AΔNS1 TCPs (MOI = 1). Forty-eight hours post-infection, cell lysates clarified by centrifugation were used for immunoprecipitation with HA-affinity agarose beads and eluates (IP) or whole cell lysates (Input) analyzed by western-blotting using antibodies specified on the right of each panel. Numbers on the left refer to molecular weight standards given in kDa. (TIF) [file ppat.1005277.s004.tif]

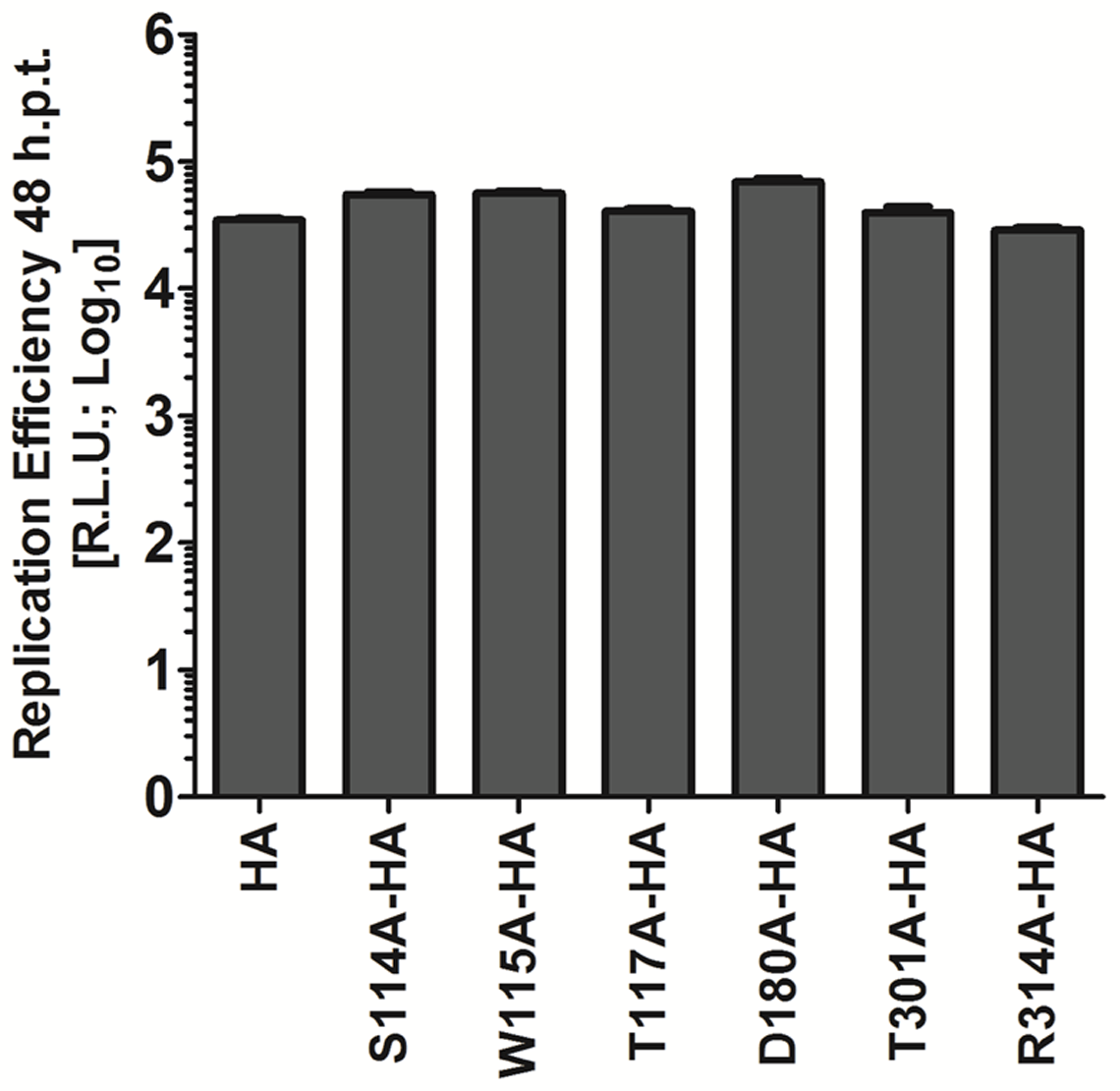

Supplement: S5 Fig — Naïve VeroE6 cells stably expressing wild-type NS1HA (HA) or different HA-tagged NS1 mutants were infected with 1 MOI of DVR2AΔNS1 TCPs. Forty-eight hours later luciferase activity was measured in the lysates to determine replication efficiency. (TIF) [file ppat.1005277.s005.tif]

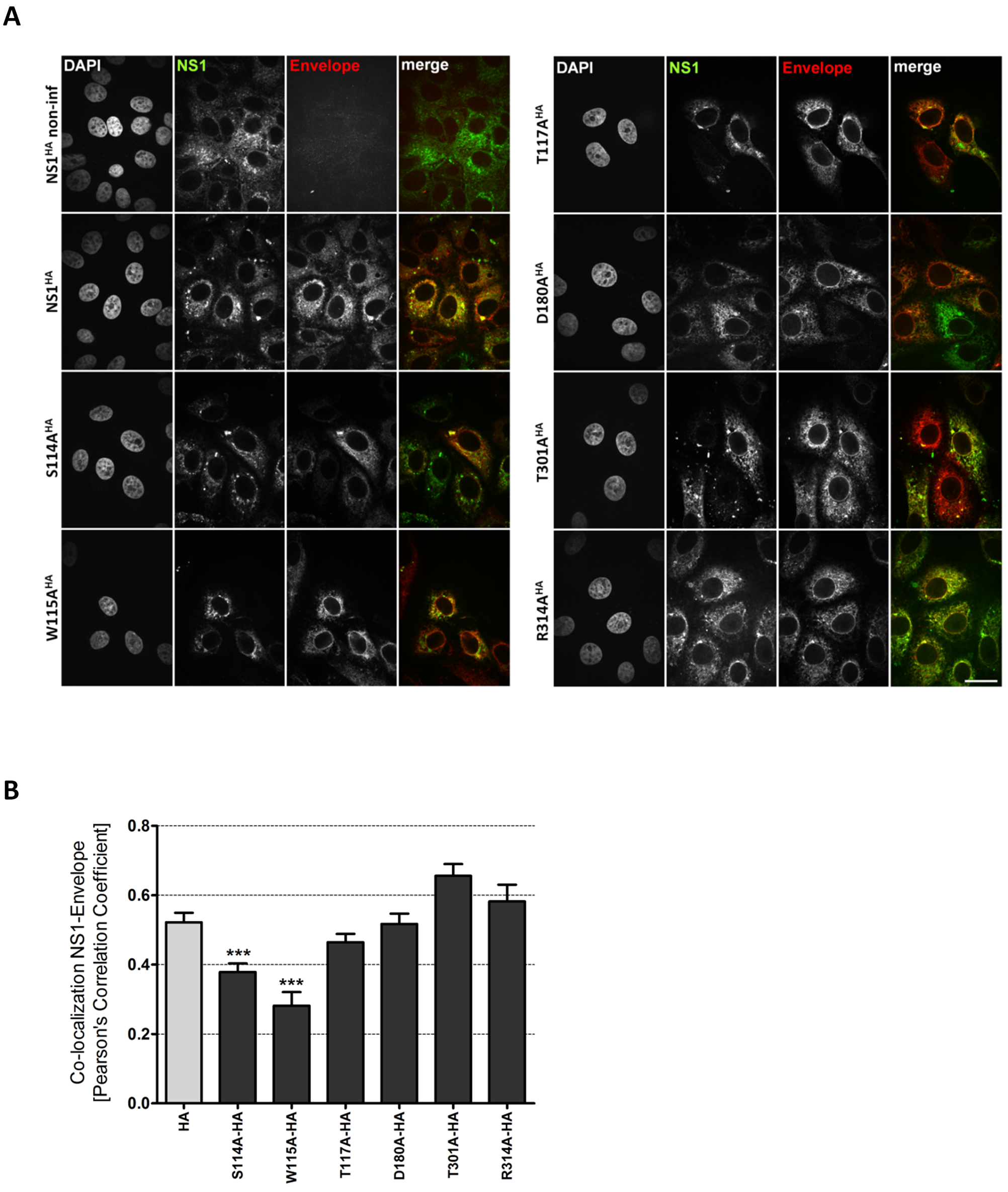

Supplement: S6 Fig — (A) Subconfluent VeroE6_NS1HA cells (NS1HA) or VeroE6 helper cells stably expressing different NS1HA mutants specified on the left of each panel, were infected with 1 MOI of DVR2AΔNS1 or mock infected (non-inf). Forty-eight hours later, cells were fixed and immunostained with rabbit HA- and mouse Envelope-specific antibodies. Scale bar represents 10 μm. (B) Co-localization of NS1 and E in the experiments shown in panel A was assessed by using the coloc2 plug-in within the Fiji (ImageJ) software package. Values represent mean and standard errors of Pearson’s correlation coefficients from at least 25 individual cells per condition. ***, P < 0.001. (TIF) [file ppat.1005277.s006.tif]
